# Supplementary material for: The mutational landscape of Bacillus subtilis conditional hypermutators shows how proofreading skews DNA polymerase error rates
Source: Nucleic Acids Res. 2025 Mar 8;53(5):gkaf147. doi: 10.1093/nar/gkaf147 (PMC11890065; doi:10.1093/nar/gkaf147)
Supplement: gkaf147_Supplemental_Files [file gkaf147_supplemental_files.zip › Tanneur-SI_NAR_revised.pdf]

Supplementary Methods and Results for

The mutational landscape of *Bacillus subtilis* conditional hypermutators shows how proofreading skews DNA polymerase error rates

Ira Tanneur[1,2], Etienne Dervyn[1], Cyprien Guérin[2], Guillaume Kon Kam King[2], Matthieu Jules[1,‡] and Pierre Nicolas[2,‡]

Provided in two separate xlsx files:

- Table S4. List of all substitutions (Table S4.1) and indels (Table S4.2) in MA-lines.
- Table S7. Substitution rates by replication-oriented triplet in *B. subtilis* (Table S7.1) and *E. coli* (Table S7.2).

Table of content

1 Supplementary Methods and Results....3

1.1 Bayesian estimation and model assessment....3

1.2 Mathematical modeling of MMR saturation....5

1.3 Algebraic analysis of a general model with 2 repair pathways and 2 error subclasses....7

1.4 Proofreading and MMR correct indels introduced by the polymerase activity of PolC....9

1.5 Genetic instability of inducible hypermutating circuits during MA experiments....10

1.6 The hypothesis of a 'ceiling' on mutagenesis can be dismissed under our conditions...11

1.7 Disentangling the effects of replication and transcription on mutation profiles....12

1.8 Quantifying the dispersion of substitution rates using robust estimation of KL divergence12

2 Supplementary Figures....14

2.1 Figure S1....14

2.2 Figure S2....15

2.3 Figure S3....16

2.4 Figure S4....17

2.5 Figure S5....18

2.6 Figure S6....19

2.7 Figure S7....20

2.8 Figure S8....21

2.9 Figure S9....22

2.10 Figure S10....23

2.11 Figure S11....24

2.12 Figure S12....25

2.13 Figure S13....26

2.14 Figure S14....27

2.15 Figure S15 28
2.16 Figure S16 29
2.17 Figure S17 30
2.18 Figure S18 31
2.19 Figure S19 32
2.20 Figure S20 33
2.21 Figure S21 34
2.22 Figure S22 35
3 Supplementary Tables 36
3.1 Table S1 36
3.2 Table S2 37
3.3 Table S3 38
3.4 Table S5 39
3.5 Table S6 40
Supplementary References 41

1 Supplementary Methods and Results

1.1 Bayesian estimation and model assessment

All models were fitted using JAGS (Plummer, 2003) version 4.3.0 for posterior sampling, through the R package rjags (https://cran.r-project.org/package=rjags). JAGS is a very popular and well-tested software for sampling for posterior distributions, it takes as input a model description and data, then it automatically builds a Monte Carlo Markov Chain sampler for the corresponding posterior distribution. It applies a Gibbs sampling strategy, considering the conditional distributions for each parameter and either selecting from a list of efficient samplers (if available) or using a Metropolis-Hastings sampler or a slice-sampler. The rest of this paragraph contains algorithmic details about the Monte Carlo Markov Chain algorithm which allow reproducing our analysis. We used a random initialisation and discarded as burn-in the first 4,000 iterations. Convergence was assessed using the Gelman-Rubin statistic (Gelman and Rubin, 1992) using two independent Monte Carlo Markov Chains. The total number of iterations was determined automatically using the package runjags (https://cran.r-project.org/package=runjags). The package uses at least 10,000 iterations, then extends the chain until the Gelman-Rubin statistics is below 1.05 for all parameters. For all cases considered, 10,000 iterations appeared sufficient to reach convergence. The chains were thinned to obtain a final size of 4,000 samples from the posterior distribution for each chain.

Posterior predictive checking to assess the fit of a model

To rely on inferences obtained from a probabilistic model, the model should fit the data reasonably well, i.e. the data should at least look plausible under the model. In the Bayesian context, posterior predictive checking (Gelman et al, 2014) recommend comparing observed data and replication data following the posterior predictive distribution, i.e. comparing observed mutation counts of type i for strain s, denoted $m_{s,i}$, to what would be expected under the posterior:

$$p(\tilde{m}_{s,i}|m_{1,1},\ldots,m_{S,I}) = \int p(\tilde{m}_{s,i}|\theta)p(\theta|m_{1,1},\ldots,m_{S,I})d\theta \quad \text{(Equation 1)}$$

where S is the number of strains, I is the number of mutation types, θ corresponds to all the model parameters, $p(.|\theta)$ is the probabilistic model (i.e. the likelihood) and $p(\theta|m_{1,1},\ldots,m_{S,I})$ is the posterior distribution.

To compare the various strains and mutation types more easily, we consider the posterior predictive distribution on an “effective mutation rate”, defined as

$$\hat{\mu}_{s,i} = \frac{m_{s,i}}{n_i t_s}$$

$$\tilde{\mu}_{s,i} = \frac{\tilde{m}_{s,i}}{n_i t_s}$$

where n_i is the number of possible sites for mutation of type i in the genome, t_s is the number of elapsed generations and $\hat{\mu}_{s,i}$ and $\tilde{\mu}_{s,i}$ can be interpreted as the rate of mutation of type i in strain s per site per generation.

We approximate the posterior predictive distribution by Monte Carlo sampling, using all iterations from the Monte Carlo Markov Chain sampler described in the previous section to estimate the mean of $\tilde{\mu}_{s,i}$, its median and the percentiles necessary to build 50% and 95% credible intervals.

Model without saturation

We introduce a simple model for mutations which does not include the saturation mechanism. This is an unconstrained model, in the sense that conditional on hyperparameters, the mutation rates for each strain are assumed to be independent. Contrary to the model with saturation in the next section, there are no biological assumptions, no mechanism introducing a dependence between the strains, the aim of this model is to infer and compare the mutation rates for the different strains taking into account the uncertainty induced by the low number of observed mutations. In details, the number of mutations (count data) of type (or context) i observed in MA-line for strain s (“wild-type” R, “MMR-deficient” MMR-, “proofreading-deficient” *C**, “MMR-deficient and proofreading-deficient” *LC**) are modelled as independent Poisson distributed random variable with mean $n_i \mu_{s,i} t_s$, *i.e.*

$$m_{s,i} \sim \text{Poisson}\left(n_i \mu_{s,i} t_s\right),$$

where n_i is the number of possible sites for mutation of type i in the genome, $\mu_{s,i}$ is the rate of mutation of type i in strain s per site per generation, and t_s is the number of elapsed generations. n_i and t_s are introduced so that $\mu_{s,i}$ can be interpreted as a mutation rate per site and per generation, which will be comparable over sites and strains, as mentioned in the posterior predictive check subsection.

The mutation rates are modelled as conditionally independent log-normal random variables

$$\mu_{s,i} \sim \log_{10} \text{Normal}(\nu_s, \sigma_s),$$

where ν_s and σ_s are strain-specific hyperparameters corresponding respectively to the location parameter and the scale parameter of the lognormal distribution (mean and standard-deviation of the normal distribution). This structure corresponds to an assumption of exchangeability among the strains, the Bayesian analog to the *independent and identical distribution* assumption of frequentist statistics, but exchangeability is a weaker assumption (it is implied by the former). ν_s and σ_s are random-variables drawn from the weakly informative hyperpriors:

$$\begin{aligned} \nu_s &\sim \text{Normal}(\text{mean} = -8, \text{std} = 3) \\ \frac{\sigma_s}{3} &\sim \text{Student}^+(\text{location} = 0, \text{scale} = 3, \text{degrees of freedom} = 5) \end{aligned}$$

Student^+ denotes the Student distribution truncated to positive values. This choice of a truncated heavy-tailed Student's t-distribution, suggested in (Gelman, 2006), allows shrinkage if compatible with the data, and is intended to be more robust than the classical inverse-Gamma prior.

This is a hierarchical structure, where ν_s represents the mean of the mutation rates (over mutation types) for strain s, and σ_s represents the variability of the mutation rates between mutation types for that strain. These two values characterise the average tendency and the dispersion of the mutation rates. These are also depicted on **Figure 5B**.

A crucial advantage of the hierarchical structure is the regularisation induced on the mutation rates, which allows including and visualising mutation rates when no mutations are observed. In the frequentist i.i.d context, for 0 observed mutations the maximum likelihood estimate would be 0, and representing this estimate on the log scale would require adding artificial counts. The hierarchical

structure can be understood as inducing a prior distribution on mutation rates. This results in a posterior distribution for plausible mutation rates covering the positive real line, which can be graphically represented without arbitrary artificial counts. This could also be obtained by adding a direct prior on each mutation rate, but with the hierarchical structure the induced prior automatically adapts to the variability among mutation types observed for each particular strain.

R package implementation of the Bayesian methodology used in this study

We built a R package available to anyone willing to analyse data from Mutation-Accumulation experiment in the same manner, available at the following link:

https://github.com/konkam/MutAccExperiments.

This R package implements estimation of the models with and without MMR saturation.

1.2 Mathematical modeling of MMR saturation

We now introduce a model which describes explicitly MMR saturation. Compared to the previous *unconstrained* model, the MMR saturation model assumes that the mutation rates of the different strains are related, i.e. *constrained* by each other. The model includes an explicit parameter for the saturation mechanism, which would be equal to 0 in the absence of saturation. Therefore, this parameter quantifies both the evidence for the existence of a saturation mechanism and its magnitude.

In practice, for each context i we assume that there is a baseline error rate γ_i. These mutations may be corrected by the Proofreading mechanism with a probability $1 - q_{\mathrm{proofreading},i}$, and further corrected by the MMR mechanism with a probability $1 - q_{\mathrm{MMR},i}$. Therefore, in the wild-type strain, the number of observed mutations is impacted by the error rate and the two repair mechanisms. This error rate is observed directly for the LC* mutant for which both MMR and Proofreading inactivated. The strain MMR- has the baseline error rate moderated by the Proofreading mechanism, the strain C* has the baseline error moderated by the MMR mechanism which can saturate. Saturation is modelled as follows: a proportion $(1 - \theta)$ of mutations are corrected by the MMR mechanism with probability $1 - q_{\mathrm{MMR},i}$, until saturation may occur. At this point, the remaining θ proportion of mutations remain uncorrected. As mentioned above, if $\theta = 0$ there is no saturation, and an estimate of θ significatly different from 0 provides evidence for saturation. This results in the following system of equations, for the rate of mutation of type i per site per generation in the four strains:

$$
\begin{aligned}
\mu_{\mathrm{LC}^*,i} &= \gamma_i \\
\mu_{\mathrm{MMR}^-,i} &= \gamma_i q_{\mathrm{proofreading},i} \\
\mu_{\mathrm{C}^*,i} &= \gamma_i \left(\theta + (1-\theta)\, q_{\mathrm{MMR},i}\right) \\
\mu_{\mathrm{wt},i} &= \gamma_i q_{\mathrm{proofreading},i} q_{\mathrm{MMR},i},
\end{aligned}
$$

where θ is a mixture parameter common to all values of i, γ[i] is the error rate before correction by proofreading or MMR, $q_{\mathrm{proofreading},i}$ is the rate at which the errors of type i escape proofreading correction, and $q_{\mathrm{MMR},i}$ is the rate at which the errors of type i escape MMR correction.

Bayesian estimation of the model with MMR saturation in the strain C*

A uniform prior on the interval [0,1] is used for θ, which assumes that the saturation may equally be large or small a priori.

For $\mu_{\mathrm{LC}^*,i}$, $\mu_{\mathrm{MMR}^-,i}$, and $\mu_{\mathrm{wt},i}$ this is the exact same lognormal model as described above in the context of the model without saturation of the MMR.

Rearranging the set of equations for the mutation rates, we can write $\mu_{\mathrm{C}^*,i}$ as

$\mu_{\mathrm{C}^*,i} = \mu_{\mathrm{LC}^*,i}\left(\theta + (1-\theta)\,\frac{\mu_{\mathrm{wt},i}}{\mu_{\mathrm{MMR}^-,i}}\right)$, which clearly illustrates that the model with saturation is more constrained (in the sense that it has fewer parameters) than the other.

Proportion of the observed mutations in the strain C* that were not subjected to MMR correction

The parameter θ corresponds to the proportion of errors which occurred in a context of saturated MMR in the strain *C**. Because these errors are never corrected by the MMR they account for a large proportion of the observed mutations in this strain. Rather than looking directly at θ, it is illustrative to observe the proportion of mutation resulting from the saturation out of all the mutations retained. This proportion, represented in the insert plot of **Figure 7A,** is given by

$\theta/(\theta + (1-\theta)q_{\mathrm{MMR},i}),$

where $q_{\mathrm{MMR},i} = \mu_{\mathrm{wt},i}/\mu_{\mathrm{MMR}^-,i}$.

Relationship between θ and the maximum number of errors that the MMR can handle

We can estimate the maximum number of errors that the MMR can handle by considering its relationship to the parameter θ. Suppose that λ corresponds to the maximum number of errors that can be treated by the MMR in one replication of the bacterial chromosome and that the number of errors submitted to the correction by the MMR follows a Poisson distribution with mean κ, then the proportion of errors that are not subjected to MMR correction because they occur after saturation writes, as a function of κ and λ:

$\eta(\kappa,\lambda) = (\kappa q_\kappa(\lambda-1) - \lambda q_\kappa(\lambda))/\kappa$,

where q_κ denotes the tail function of the Poisson distribution of mean κ, *i.e.* $q_\kappa(l) = 1 - \sum_{n\le l}\exp(-\kappa)\kappa^n/n!$.

To understand the origin of this relationship, note that the mean of the Poisson distribution truncated to $n > \lambda$ writes $\kappa q_\kappa(\lambda-1)/q_\kappa(\lambda)$, where κ is the mean of the non-truncated distribution. Thus, in a draw over λ of the Poisson distribution with mean κ, the average of errors over λ is $\kappa q_\kappa(\lambda-1)/q_\kappa(\lambda) - \lambda$. Multiplied by $q_\kappa(\lambda)$, the probability to have a draw over λ, and divided by κ the total number of errors, this gives the formula for the proportion of errors $\eta(\kappa,\lambda)$ reported above.

In strain *C**, $\kappa = \sum_i \gamma_i n_i$ and thus θ correspond to the proportion of errors which occurred in a context of saturated MMR in the strain *C*.* It writes $\theta = \eta\left(\sum_i \gamma_i n_i, \lambda\right)$, which can be inverted numerically to obtain λ as a function of θ and $\sum_i \gamma_i n_i$(as represented in **Figure 7B**). Thanks to the

relation between λ and θ, the estimated value of θ then allows estimating λ, the maximum number of errors that the MMR can handle.

Of note, in the wild-type, the mean number of errors submitted to the correction by the MMR is $\kappa = \sum_i \gamma_i n_i q_{\text{proofreading},i}$, which corresponds to $\kappa = \sum_i \mu_{\text{MMR}^-,i} n_i$ and we can verify that $\eta(\kappa, \lambda)$ is very close 0, with the mutation rates observed in the experimental data (there is a low proportion of errors caused by saturation of MMR in the wild-type).

Relationship between θ and the distance the polymerase must travel after a first error before MMR can handle a second error.

An alternative mechanism can lead to MMR saturation: the requirement of a minimum distance between two errors corrected by the MMR. This minimum distance is hereafter denoted by R in analogy with the refractory period of a neuron. Assuming that polymerase errors occur as a Poisson process with rate γ, the errors subject to MMR correction follow a renewal process with inter-event distance R+1/γ. The average ratio between the number of events subject to MMR correction and the total number of errors, which corresponds to 1-θ, is then 1/(1+γR).

Thus, we can derive R from μ and θ using the inverse relationship R=(1/γ).[θ/(1-θ)].

Considering $\gamma = 7.4\times10^{-7}$ bp^{-1} (the total point mutation rate in *LC**) and the point estimates of θ between 0.071 and 0.084 (depending on the data set, estimated in *C**), we obtain a value of R between 103 and 124 kbp.

1.3 Algebraic analysis of a general model with 2 repair pathways and 2 error subclasses

In this section, we describe a general model that considers aggregated subclasses of mutations and arbitrary correction rates. This model explains the conditions under which we can expect to observe super-multiplicative or sub-multiplicative effects of the error correction mechanisms. More precisely, we consider here a model with two subclasses of errors, numbered 1 and 2, and in which the wild-type possess two error correction pathways, denoted a and b. The mutation rates are denoted $\mu_{a,b}$ for the wild-type, $\mu_{-,b}$, $\mu_{a,-}$, and $\mu_{-,-}$, for pathways a and b deactivated separately or simultaneously. In this model the mutation rates write

$$\begin{aligned}
\mu_{-,-} &= \gamma_1 + \gamma_2 \\
\mu_{a,-} &= \gamma_1 q_{a,1} + \gamma_2 q_{a,2} \\
\mu_{-,b} &= \gamma_1 q_{b,1} + \gamma_1 q_{b,2} \\
\mu_{a,b} &= \gamma_1 q_{a,1} q_{b,1} + \gamma_2 q_{a,2} q_{b,2},
\end{aligned}$$

where $\gamma_1 \geq 0$ and $\gamma_2 \geq 0$ correspond to the initial rates of both subclasses of errors, $q_{a,1} \in (0,1)$ and $q_{a,2} \in (0,1)$ are the rates at which the errors of subclass 1 and 2, resp., escape correction by pathway a, and $q_{b,1} \in (0,1)$ and $q_{b,2} \in (0,1)$ are the rates at which the errors of subclass 1 and 2, resp., escape correction by pathway b.

This model has 6 free parameters and can fit perfectly any set of four mutation rates satisfying the four "natural" order relationships $\mu_{-,-} \geq \mu_{a,-}, \mu_{-,-} \geq \mu_{-,b}, \mu_{a,-} \geq \mu_{a,b}$, and $\mu_{-,b} \geq \mu_{a,b}$, and the condition that the increments in mutation rates are at least additive, which can be written

$\mu_{-,-} \geq \mu_{a,-} + \mu_{-,b} - \mu_{a,b}$ or $\mu_{-,-} \geq (\mu_{a,-} - \mu_{a,b}) + (\mu_{-,b} - \mu_{a,b}) + \mu_{a,b}$.

In practice, to find a set of parameters which fit a particular set of mutation rates we can for instance choose $q_{a,1} = 0$ and $q_{a,2} = 1$ (pathway 'a' corrects perfectly the errors of subclass 1 and none of subclass 2) and then identify

$$\gamma_1 = \mu_{-,-} - \mu_{a,-}$$

$$\gamma_2 = \mu_{a,-}$$

$$q_{b,1} = \frac{\mu_{-,b} - \mu_{a,b}}{\mu_{-,-} - \mu_{a,-}}$$

$$q_{b,2} = \frac{\mu_{a,b}}{\mu_{a,-}}$$

Special cases

In the case with a single subclass of error ($\gamma_1 = 0$ or $\gamma_2 = 0$), then

$$\frac{\mu_{-,-}}{\mu_{a,b}} = \frac{\mu_{a,-}}{\mu_{a,b}} \frac{\mu_{-,b}}{\mu_{a,b}}.$$

We can see in this equation the ratios by which the mutation rates increase with the deactivation of each correction pathway are multiplicative.

The same property of multiplicative ratios holds in another special case where one pathway does not distinguish the two subclasses of errors ($q_{a,1} = q_{a,2}$ or $q_{b,1} = q_{b,2}$).

On the contrary, if each pathway corrects only a specific subclass of error (e.g. $q_{a,2} = 1$ and $q_{b,1} = 1$ meaning that 'a' cannot correct subclass 2 and 'b' cannot correct subclass 1), then

$$\mu_{-,-} - \mu_{a,b} = (\mu_{-,b} - \mu_{a,b}) + (\mu_{a,-} - \mu_{a,b}).$$

In this case the increments of the mutation rates associated with the deactivation of each correction pathway are additive.

Extreme scenarios of super-multiplicative and sub-multiplicative ratios

The condition for multiplicative ratios is

$$\frac{\mu_{-,-}}{\mu_{a,b}} = \left(\frac{\mu_{a,-}}{\mu_{a,b}}\right) \cdot \left(\frac{\mu_{-,b}}{\mu_{a,b}}\right),$$

and can be rewritten

$$R = \frac{\mu_{-,-}\mu_{a,b}}{\mu_{a,-}\mu_{-,b}} = 1,$$

with $R > 1$ corresponding to "super-multiplicative" ratios and $R < 1$ corresponding to "sub-multiplicative" ratios.

To explore algebraically how to obtain extreme values of R, this ratio can be written in terms of the 6 model parameters

$$R = \frac{\gamma_1^2 q_{a,1} q_{b,1} + \gamma_1\gamma_2 q_{a,1} q_{b,1} + \gamma_1\gamma_2 q_{a,2} q_{b,2} + \gamma_2^2 q_{a,2} q_{b,2}}{\gamma_1^2 q_{a,1} q_{b,1} + \gamma_1\gamma_2 q_{a,1} q_{b,2} + \gamma_1\gamma_2 q_{a,2} q_{b,1} + \gamma_2^2 q_{a,2} q_{b,2}}.$$

We note that R does not change if γ_1 and γ_2 are multiplied by a same constant, or $q_{a,1}$ and $q_{a,2}$ are multiplied by a same constant, or $q_{b,1}$ and $q_{b,2}$ are multiplied by a same constant. Without loss of generality, for the search of the extreme values of R, we can then set $\gamma_1 = \gamma$ and $\gamma_2 = 1-\gamma$, $q_{a,1} = \alpha$ and $q_{a,2} = 1-\alpha$, and $q_{b,1} = \beta$ and $q_{b,2} = 1-\beta$. The ratio R can then be rewritten as a function of γ, α, β as

$$R(\gamma,\alpha,\beta) = \frac{\gamma\cdot\alpha\cdot\beta + (1-\gamma)\cdot(1-\alpha)\cdot(1-\beta)}{(\gamma\cdot\alpha + (1-\gamma)\cdot(1-\alpha))\cdot(\gamma\cdot\beta + (1-\gamma)\cdot(1-\beta))}.$$

To find the extrema of this function $R\left(\gamma,\alpha,\beta\right)$ we can do a log-transformation (monotonic) and study the first order partial derivatives $\partial \log R/\partial\alpha$, and $\partial \log R/\partial\beta$. The partial derivative $\partial \log R/\partial\alpha$ is null if and only if $\gamma(1-\gamma)(2\beta-1) = 0$ and has the sign of $2\beta - 1$. Symmetrically, the partial derivative $\partial \log R/\partial\beta$ is null if and only if $\gamma(1-\gamma)(2\alpha-1) = 0$ and has the sign of $2\alpha - 1$

We deduce from these derivatives that the ratio is constant over the values of (α,β) when $\gamma = 0$ or $\gamma = 1$, which correspond to a scenario with a single non-empty subclass. We have already pointed out that the property of multiplicative ratio holds in this special case ($R(0,\alpha,\beta) = 1$ and $R(0,\alpha,\beta) = 0$).

We also see from these derivatives that for any given value of γ, the ratio is constant over the values of α when $\beta = 1/2$ and reciprocally over the values of β when $\alpha = 1/2$. We have already pointed out that the property of multiplicative ratio holds in this special case ($R(\gamma,\alpha,1/2) = 1$ and $R(\gamma,1/2,\beta) = 1$).

By examining the sign of the two first order partial derivatives $\partial \log R/\partial\alpha$ and $\partial \log R/\partial\beta$ we see that for any given value of γ such as $0 < \gamma < 1$, the ratio increases when $(\alpha,\beta) \to (1,1)$ (or symmetrically $(\alpha,\beta) \to (0,0)$) and decreases when $(\alpha,\beta) \to (1,0)$ (or symmetrically $(\alpha,\beta) \to (0,1)$). The corresponding extrema can be computed and are $R(\gamma,0,1) = 0$ and $R(\gamma,1,1) = 1/\gamma$ (symmetrically $R(\gamma,1,0) = 0$ and $R(\gamma,1,1) = 1/(1-\gamma))$.

The minimum value of R is 0 and is reached in a special case of additive increments in which the two repair systems are both complementary and perfectly efficient $\mu_{a,b} = 0$.

The maximum value of R for $0 < \gamma < 1$ is $1/\gamma$ if $\gamma < 1/2$ and $1/(1-\gamma)$ if $\gamma > 1/2$. In this scenario the two repair pathways correct the same subclass of error and R can be increased indefinitely by decreasing the size (γ or $(1-\gamma)$) of the subclass of error which are not corrected by any of the two considered pathways. In this way the model can account for arbitrary super-multiplicative ratios.

1.4 Proofreading and MMR correct indels introduced by the polymerase activity of PolC

We examined the accumulation of short insertions and deletions in our MA-lines. Although the small number of indels compared to substitutions makes it more difficult to detect statistically significant changes, the MA-lines that showed the largest decreases in substitution rates (LC^*_1 and LC^*_4) also showed decreases in indel rates (**Figure S6**).

Out of the 222 insertions and 232 insertions found in our MA-lines after discarding the intervals in which a decrease in substitution rate was detected, only 1 insertion and 3 deletions were 2 bp in length, all others were of length 1 bp. This differs from that reported for R^{3610} (Sung et al. 2016) where

(after mapping to our reference subset of positions in *B. subtilis* 168 for consistency with our data) 35 of the 88 deletions and 4 of the 24 insertions were of length > 2 bp (the largest deletion being of length 4,136 bp, **Table S4**). We noted that the start or entirety of some of the long deletions found in the R^{3610} strains were identical to the start of the immediately following sequence (e.g., deletions at positions 491,610 and 959,732), suggesting that these deletions occurred by local recombination. To compare the similar events in the different strains, we discarded any indel of length > 2 bp from our calculation of indel rates (**Table S5**).

Indel rates were lower than substitution rates in all strains (**Figure S7**). Indel rates and substitution rates exhibited a strong positive correlation (Pearson r=0.99) across strains (**Figure S7A**). This correlation is consistent with a contribution of both MMR and proofreading to the repair of substitutions and indels. Intriguingly, the strain MMR-^{168} exhibited a higher ratio of indels versus substitutions than any other strains and differed significantly from MMR-^{3610} and MMR-^{PY79} and both insertions and deletions contributed to this difference (**Figure S7B**).

All short indels except a single deletion in our MA-lines were found in homopolymers or tandem repeats of length ≥ 2. Overall, 97.6% of the short indels in our MA-lines occurred in homopolymers of length ≥ 4. These results are consistent with the model of (Streisinger et al. 1966), according to which displacement of the template strand or newly synthesized strand is more frequent in microsatellite regions, and thus indels are more frequent in these regions; with indel rates increasing with the length of the homopolymers for both insertions and deletions (**Figure S8**). In all strains, the indel rates tended to increase linearly with homopolymer length at least until length 6. A decrease in slope after length 7 was visible in MMR-^{3610}, MMR-^{PY79}, *C** and *LC**. Using a GLM (Poisson data with log-link), we modeled indel rates in the homopolymers as a linear of homopolymer length, between lengths 2 and 6. The first-order terms were not significantly different between strains *C**, *LC**, R^{3610}, and R^{PY79}, suggesting that PolC proofreading and MMR activities do not have their own biases, and they simply lower the error rate of the polymerase. Similar trends have been observed in yeast for these homopolymer lengths when MMR and/or proofreading are abolished (Lujan et al. 2015). We noticed however a difference in the initial slope when MMR-^{168} was included in the comparison (ANOVA p-value 0.00087), reflecting the steepest in MMR-^{168}.

The graphical representation of indel rates as a function of the length of the homopolymers (**Figure S8**) also revealed that the elevated indel rates in MMR-^{168} compared to all other strains, and in particular to MMR-^{3610} and MMR-^{PY79}, resulted principally from abundant indels in the longest homopolymers. Indels in homopolymers of length ≥ 8 represented 56% of the indels in MMR-^{168} against 22% in MMR-^{PY79} and 12% in MMR-^{3610}.

1.5 Genetic instability of inducible hypermutating circuits during MA experiments

Nonsynonymous mutations on the negative dominant *polC** or *mutL** alleles, or mutations on their RBS or promoter (P_{hs}), or nonsynonymous mutations on the *lacI* gene (which represses P_{hs}), as well as other mutations in untranslated regions of the *polC** and *mutL** mRNA that may alter their stability, will directly modify the mutation rate of *L**, *C**, and *LC** strains. Therefore, we established a list of the mutations identified on these genetic elements and in their vicinity (**Table S6**).

For the mutant alleles *mutL** and *polC**, reads from the native alleles (*mutL* and *polC*) were also mapped to the insert, and variant calling *per se* cannot distinguish between a mutation in the native

and mutant allele. However, at positions where bases differed from the reference on these genes in individual samples, the proportion of these alternative bases was bimodally distributed, with two peaks, located at approximately at 40% and 60% of the reads **(Figure S10**). Since the characteristic point mutations of both *polC** and *mutL** accounted for more than 50% of the reads (between 52% and 71% and between 65% and 75%, respectively) at their respective positions, we predicted that the mutations for which the majority of the reads differed from the reference would be on the mutant allele, while the others would be on the native allele. This prediction is consistent with the chromosomal position of the *amyE* locus where the mutant alleles are inserted, *i.e.* closer to the origin of replication of the chromosome than either native allele, and thus expected to be more abundant in the sample due to ongoing replication. To verify this, we used specific primers to amplify and sequence either the native or the inserted allele. Of the 5 PCR-verified mutations, all were found on the predicted allele (**Table S6**).

For lines C^*_3, LC^*_2, LC^*_3 and LC^*_4, we found nonsynonymous mutations on *polC** that could inactivate or decrease the activity of PolC*, allowing native PolC to replicate DNA better and correct mutations through its exonuclease activity. However, we did not find mutations in any of the above-mentioned genetic elements in the LC^*_1 line, and the appearance of the mutation found in LC^*_3 may be posterior to the decrease in mutation rate. This suggests the presence of compensatory mutations in the *B. subtilis* chromosome that can decrease substitution rates independently of the inducible synthetic hypermutation circuits.

In the strain *LC*,* all mutations on an inserted allele were found on *polC**, and none on *mutL**, consistent with the larger size of *polC** (4,311 bp) compared to *mutL** (1,884 bp). No mutation was found on the shorter genetic elements *lacI*, P_{hs} or RBS of the mutant alleles.

1.6 The hypothesis of a 'ceiling' on mutagenesis can be dismissed under our conditions

The use of yeast diploid cells, which are less prone to cell death due to the compensation of essential allele inactivation by their homologous counterparts, enabled the measurement of mutation rates up to 10,000-fold above wild-type levels in MMR- and proofreading-deficient mutant cells (Herr et al., 2011). However, in yeast haploid cells, the same mutants exhibited mutation rates 1,000-fold above wild-type levels, leading the authors to conclude that a "ceiling" on mutagenesis exists. Although the *LC** mutant strain exhibits a nearly 1,000-fold increase in mutagenesis compared to wild-type levels, several observations suggest that this ceiling has not been reached, (i) the growth rate remains unaffected upon induction of the hypermutator systems (**Figure S3**), (ii) mutation rates remain stable during the initial MA-line steps (**Figure S6**), and (iii) mutation rates in *LC** cells were comparable or slightly lower than those observed in *E. coli* MMR- and proofreading-deficient mutants (**Figure S10**, Niccum et al., 2016). Additionally, and in line with data obtained in yeast haploids, rapidly selected mutants in *LC** lines harbored mutations in the polymerase gene (**Table S6**).

1.7 Disentangling the effects of replication and transcription on mutation profiles

Due to the strong collinearity between transcription and replication in *B. subtilis*, apparent replication-oriented biases may involve a contribution from transcription. In fact, and as expected, the asymmetry between template and non-template (also known as coding) strands of transcription tends to be similar

to the asymmetry between leading and lagging strands of replication (compare **Figures S11A** and **4A**). To analyze whether the biases are related to replication, transcription, or both, we used a generalized linear model (GLM). This approach allowed us to disentangle the contributions of replication and transcription, and showed that once the replication strand was given, the transcription strand had no statistically significant effect on substitution rates in the reference strains (R^{3610} and R^{PY79}), in MMR^{-3610}, and in the hypermutators constructed here (MMR^{-168}, *C**, *LC**) (ANOVA *p*-value≥0.05, considering each strain separately). This is consistent with the comparison between estimates of local substitution rates distinguishing replication and transcription strands (**Figure S11B**) and also with previous statistical analyses on MMR^{-PY79} (Schroeder et al. 2016). For example, regardless of the transcription strand, the substitution rate at C sites in the MMR-deficient strains is higher on the leading strand than on the lagging strand, and the substitution at T sites in the *LC** strain is higher on the lagging strand than on the leading strand. Further examination of these rates (**Figure S11B**) does not indicate a higher substitution rate in genes encoded “head-on” (*i.e.* where the template strand of transcription is the leading strand of replication), regardless of the strain.

Regarding the location in or out of a coding region, a significant difference in mutation rates was found for the reference strains (R^{3610} and R^{PY79}) and in the MMR-deficient strains (MMR^{-3610} and MMR^{-PY79}), with the reference strains having a higher substitution rate in noncoding regions, whereas MMR-deficient strains have a higher substitution rate in coding regions (ANOVA p-value 5×10^{-8} for R^{3610} and 1×10^{-11} for MMR^{-PY79} and **Figure S11C**) as also found for MMR^{-3610} in the analysis of (Sung et al., 2015). A higher G+C-content in coding than in noncoding regions combined with a globally higher substitution rate at sites corresponding to G:C pairs, and more generally a difference in nucleotide composition between coding and noncoding regions, may lead to a higher substitution rate in coding regions. In support of this idea, we found that a GLM including “triplet” and “coding status” does not fit the data for MMR^{-3610} and MMR^{-PY79} significantly better than a GLM including only the “triplet” effect (ANOVA *p*-value>0.05). In contrast, a model including “triplet” and “coding status” fits the data for R^{3610} and R^{PY79} better (ANOVA *p*-values 2×10^{-7} and 3×10^{-4}, respectively).

The presence of the base in a coding or noncoding region could be refined using expression level data. However, the expression level of the region does not appear to affect substantially the substitution rate in any strain (**Figure S11D**).

Taken together, these results lead us to conclude that the effect of transcription is absent or very marginal under our growth conditions.

1.8 Quantifying the dispersion of substitution rates using robust estimation of KL divergence

We quantified the dispersion of stranded-triplet substitution rates in strain s by the KL divergence between the unknown categorical distribution underlying the observed counts (probability density function denoted f_s) and the known categorical distribution if the rates were uniform. The probability density function of this known distribution is denoted g and is given by

$g[i] \propto n[i]$,

where n[i] is the number of possible sites for mutation type i in the genome.

The KL divergence of f_s from g writes

$D_{KL}(f_s || g) = \Sigma_i f_s[i] \log(f_s[i]/g[i])$

$= \Sigma_i f_s[i] \log(f_s[i]) - \Sigma_i f_s[i] \log(g[i])$.

The second term can be estimated by replacing $f_s[i]$ by its maximum likelihood estimate, $f^{(ML)}{}_s[i] \propto m[s,i]$, in the sum. As a linear combination of unbiased estimators, this gives an unbiased estimate.

The first term corresponds to $-H(f_s)$, where H is the Shannon entropy which cannot be estimated using the same approach since the plug-in estimator of the Shannon entropy, $\Sigma_i f^{(ML)}{}_s[i] \log(f^{(ML)}{}_s[i])$, known to be very poor (strongly biased downwards) when the $f^{(ML)}{}_s[i]$'s are based on small counts. Instead, we used an entropy estimation method that is robust to sparse sampling (Nemenman et al. 2001).

2 Supplementary Figures

2.1 Figure S1

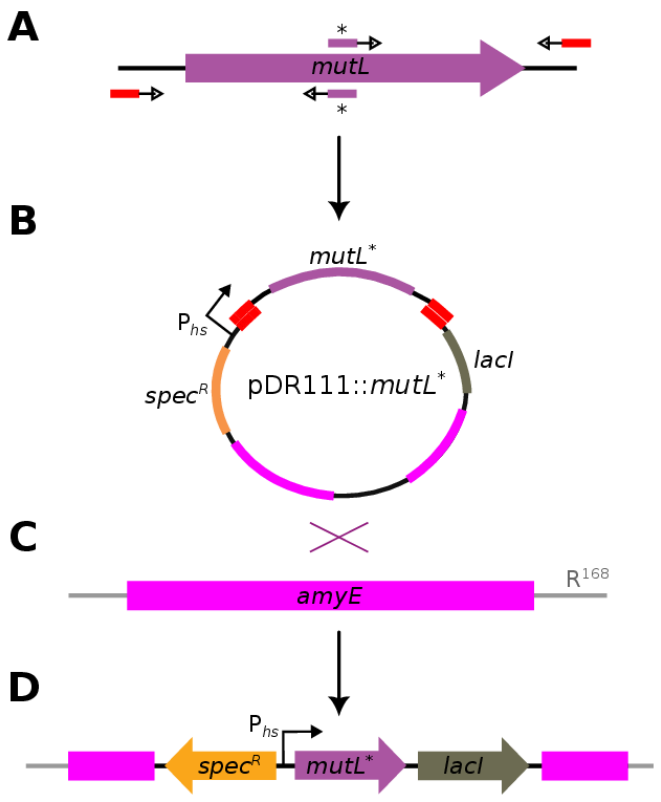

Figure S1. Steps for the introduction of a mutant allele into the chromosome of *B. subtilis* using pDR111. A. PCR amplification to introduce the desired point mutation N34H into *mutL*. **B.** Insertion into pDR111. **C.** Insertion into the *B. subtilis* genome by homologous recombination in *amyE.* **D.** Final insert in the chromosome of the strain *L**. A similar procedure was used to obtain *C** and *LC**, but with *polC mut1* amplified directly from a strain carrying this mutant allele of *polC*.

2.2 Figure S2

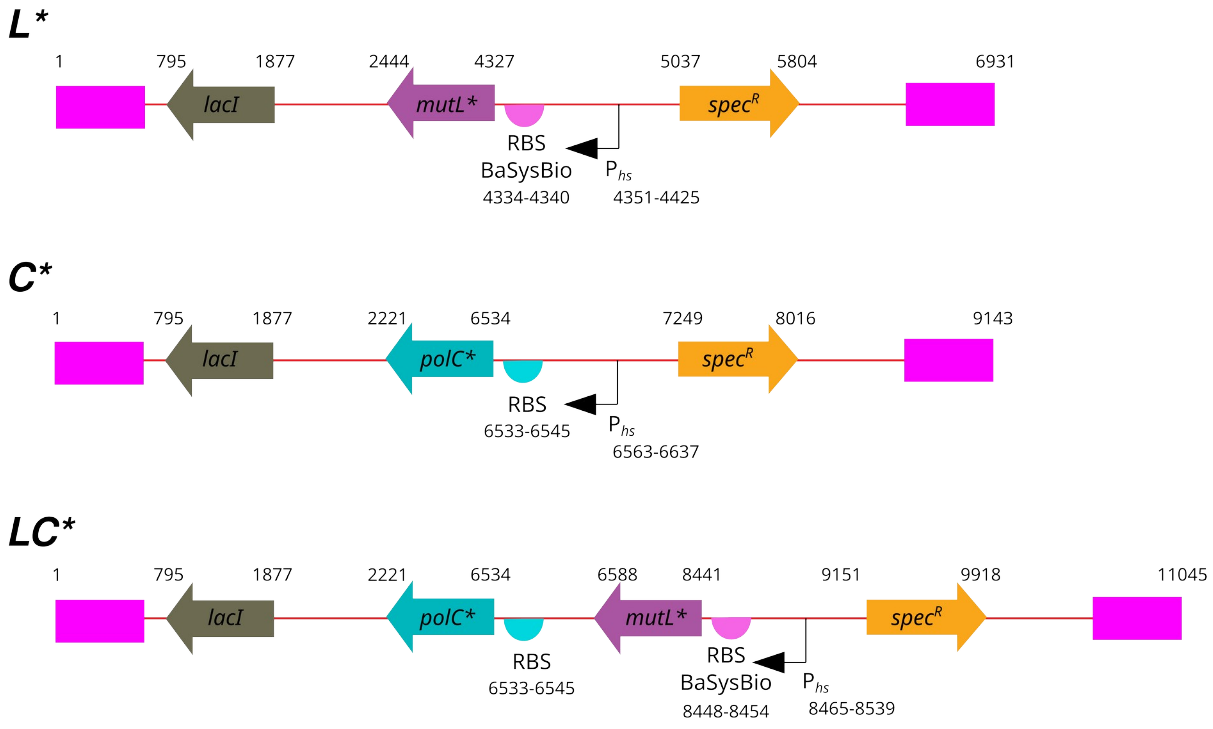

Figure S2. Schematic representation of the inserts carrying the hypermutator inducible circuits. Regions of *amyE* are represented in magenta. Coordinates of the different genes, promoters and RBSs are given in the three strain-specific inserts.

2.3 Figure S3

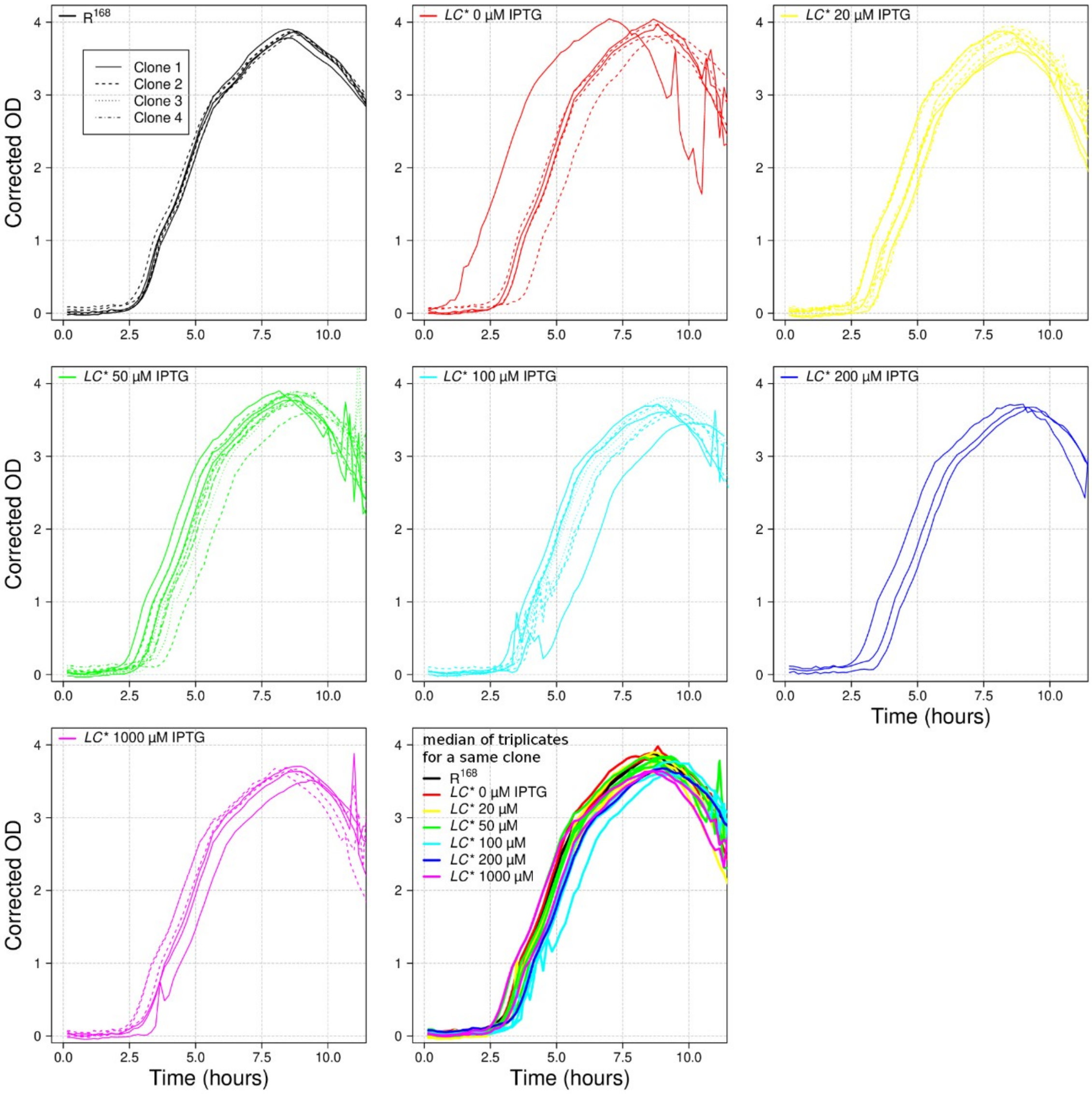

Figure S3. Growth rates of strain *LC at increasing IPTG concentrations and comparison with R^{168}.** Corrected optical density (microplate reader measured OD) at 600 nm during growth in LB at 37°C. Triplicate growth curves for several isolated clones (1 to 4) are shown for different combinations of strains and conditions in the first seven plots. In the last plot, the combinations of strains and conditions are all represented together, with each line corresponding to the median of the growth curves for the same clone.

2.4 Figure S4

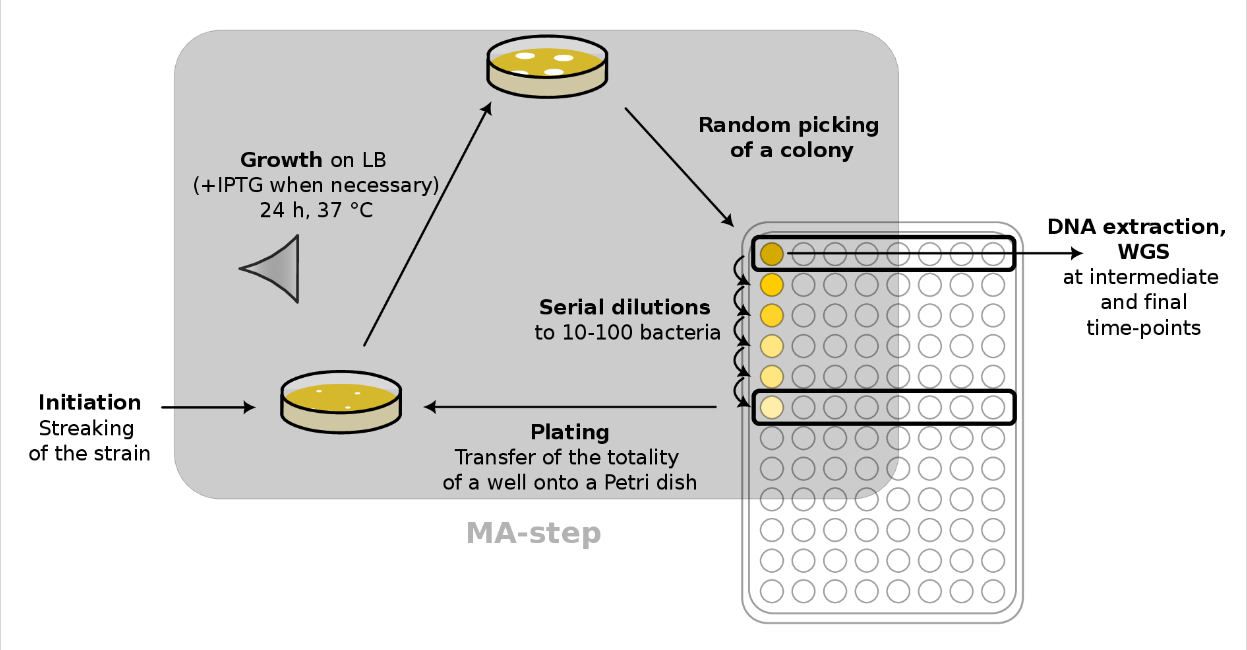

Figure S4. Mutation accumulation protocol. The use of a 96-well plaque allows parallel serial dilution of independent MA-lines. Each well in the first row of the 96-well plaque contains all the cells of a single colony; the mutations identified by sequencing are those that occurred before the clonal expansion of that colony (i.e. before the current MA-step).

2.5 Figure S5

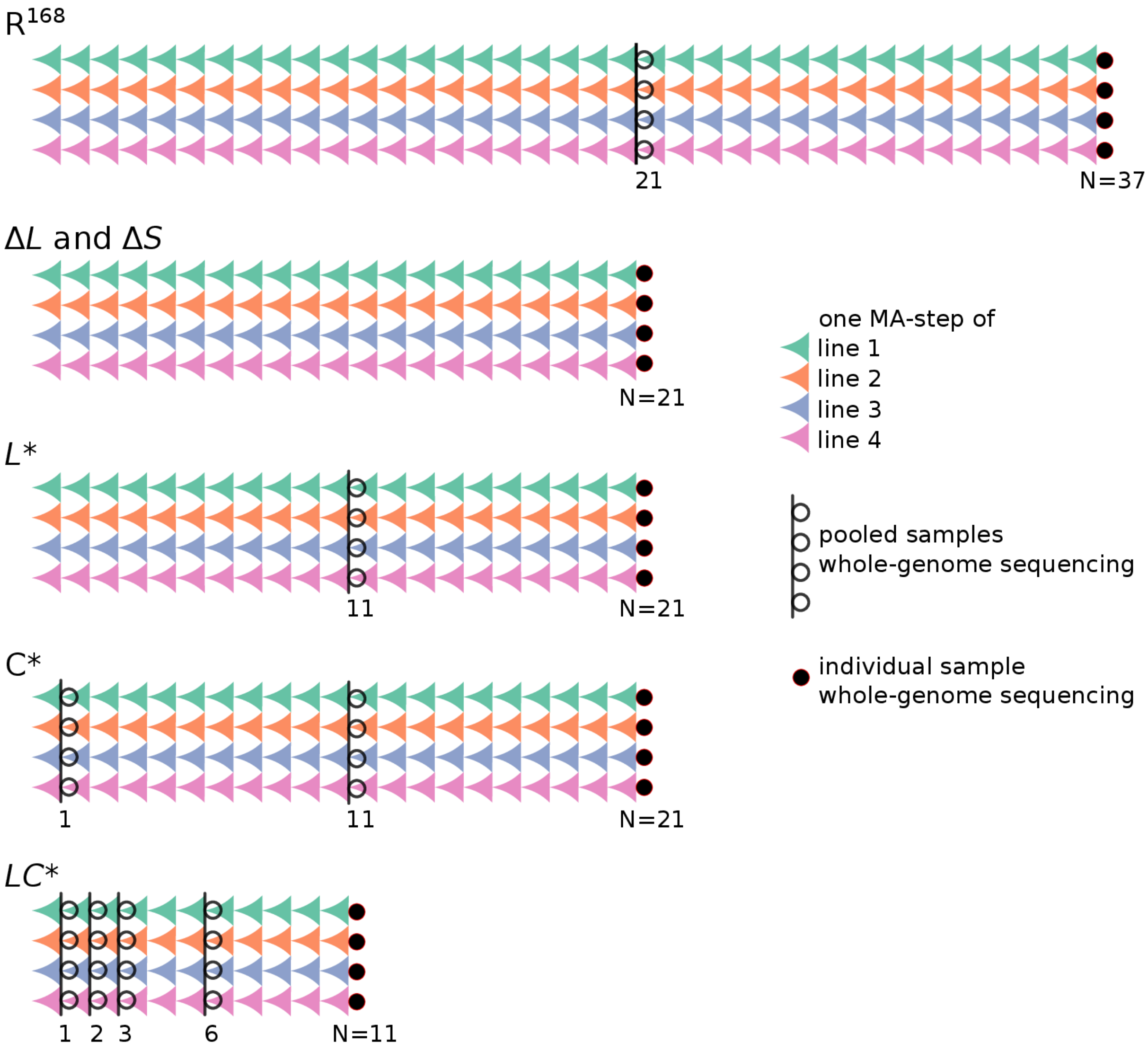

Figure S5. Design of mutation accumulation experiments. For each strain the four independent MA-lines are represented, with MA-steps symbolized by concave triangles (clonal expansion during colony growth). The total number of MA-steps (N) and the time points of whole-genome sequencing are reported. Sequencing data from pooled samples identify the time interval in which a mutation occurred.

2.6 Figure S6

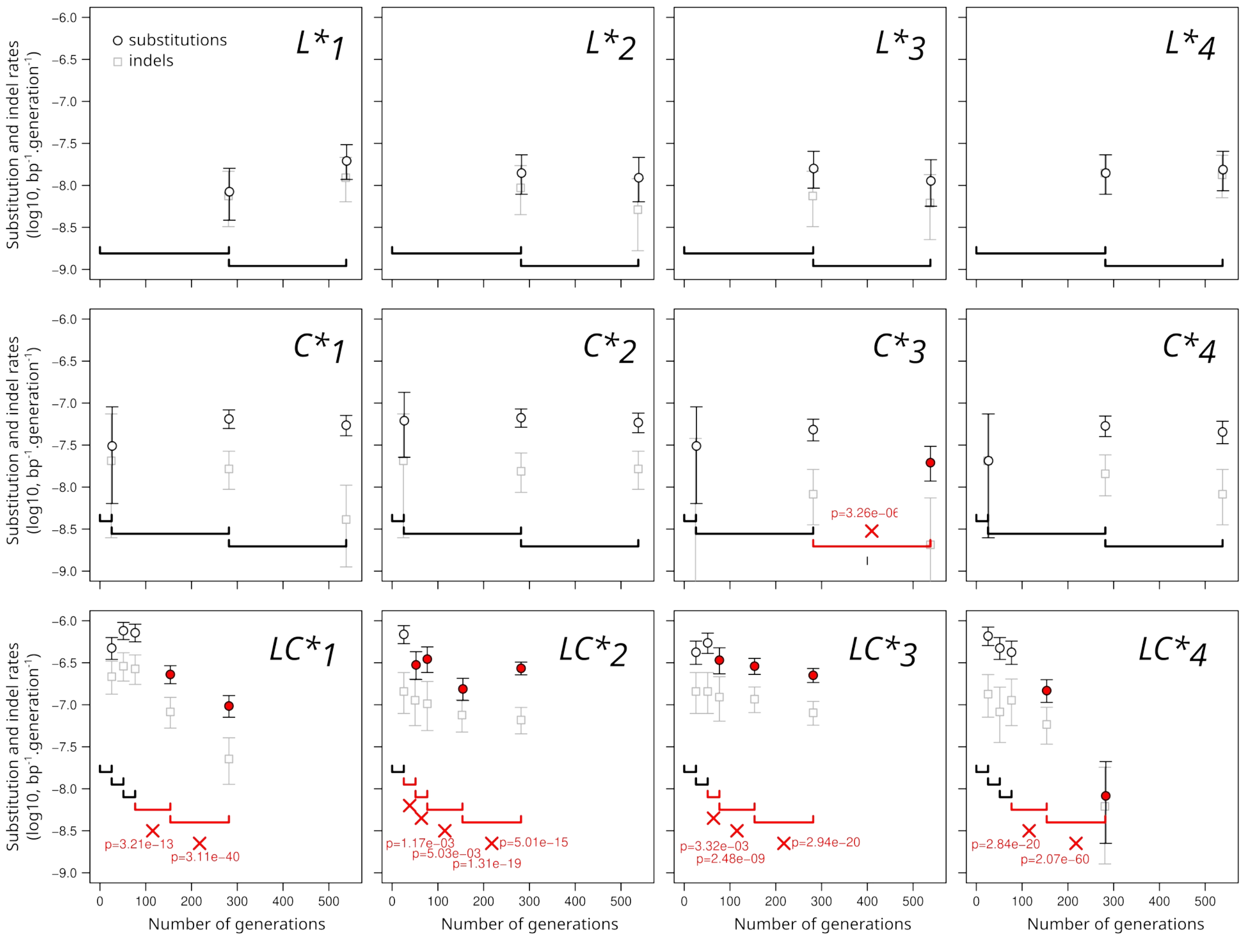

.

Figure S6. Evolution of substitution and indel rates during mutation accumulation experiments. Rates per base and per generation for the four MA lines of the *L** (top), *C** (middle) and *LC** (bottom) strains are calculated using the number of mutations fixed within each interval. Substitution and indel rates are represented by black circles and gray squares, respectively; 95% confidence intervals are represented. The time intervals discarded due to a decrease in substitution rate (p-value ≤ 0.01) are indicated with red crosses along with the associated p-values. A chi-squared test was used here to compare the number of substitutions in a given interval with the number of substitutions in previous, undiscarded intervals of the same strain (all MA lines), taking into account the number of generations.

2.7 Figure S7

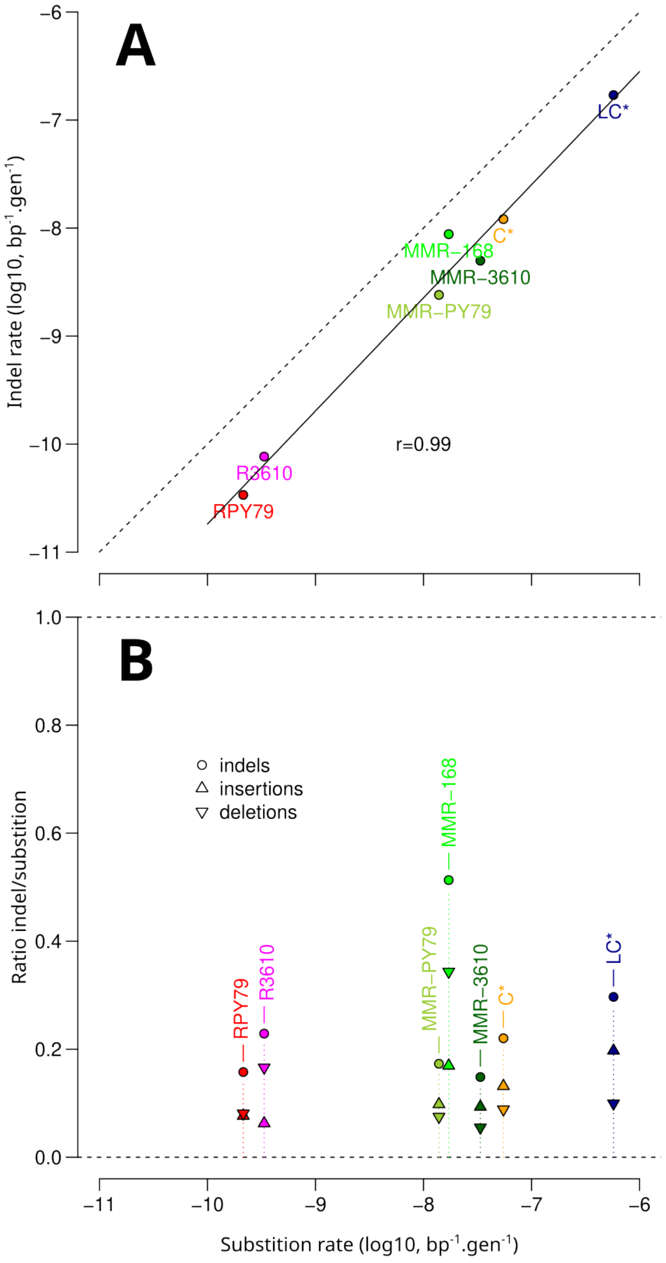

Figure S7. Comparison between indel and substitution rates across strains. A Indel versus substitution rates, both in log scale. The black line represents the fit of a linear model of log(indel rates) vs. log(substitution rates); the Pearson correlation coefficient between indel and substitution rates is 0.99 (p-value = 1.75×10^{-5}). **B** Ratio between indel (natural scale) and substitution rates across strains separated by substitution rates (log scale). The respective contributions of insertions and deletions are also shown.

2.8 Figure S8

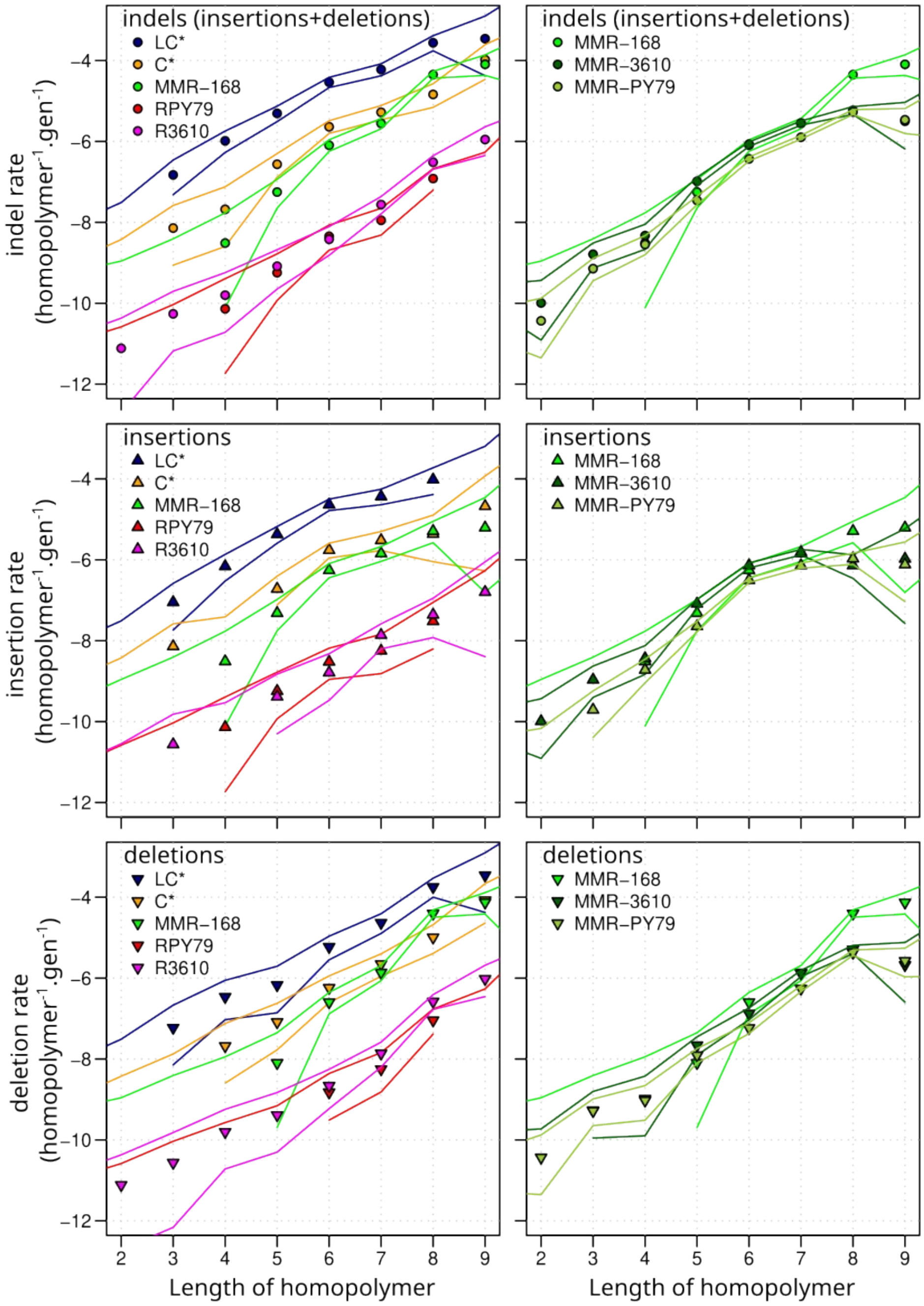

Figure S8. Rates of short insertions and deletions as a function of homopolymer length. The rate in a homopolymer of a given length is given per homopolymer occurrence and per generation. Results are shown for insertions and deletions of length ≤ 2 bp, for insertions and deletions aggregated or separated (three rows). The left column superimposes the rates across genotypes with proficient or deficient for MMR and proofreading. The right column superimposes the rates for the three MMR- datasets. The symbols (circles and triangles) represent the point estimates, the coloured lines represent the 95% confidence intervals.

2.9 Figure S9

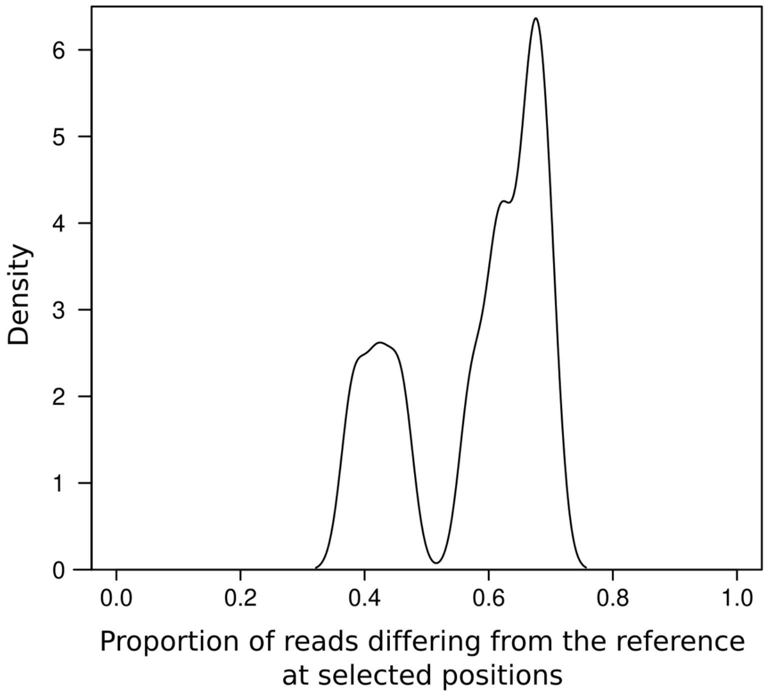

Figure S9. Bimodal distribution of the proportion of reads differing from the reference for reads mapped in the inducible synthetic circuits. Density estimation based on substitutions at the 10 positions in *mutL*, *mutL**, *polC* or *polC** listed in Table S3 with a Gaussian kernel smoother (standard deviation = 0.2). The bimodal distribution reflects a higher gene dosage for *mutL** and *polC** than for *mutL* and *polC* because the *amyE* locus where the mutant alleles are inserted is closer to the replication origin of the chromosome. Mutations associated with an alternative allele frequency above 50% are in *mutL** and *polC** as verified by PCR for some of the mutations (see **Table S4**).

2.10 Figure S10

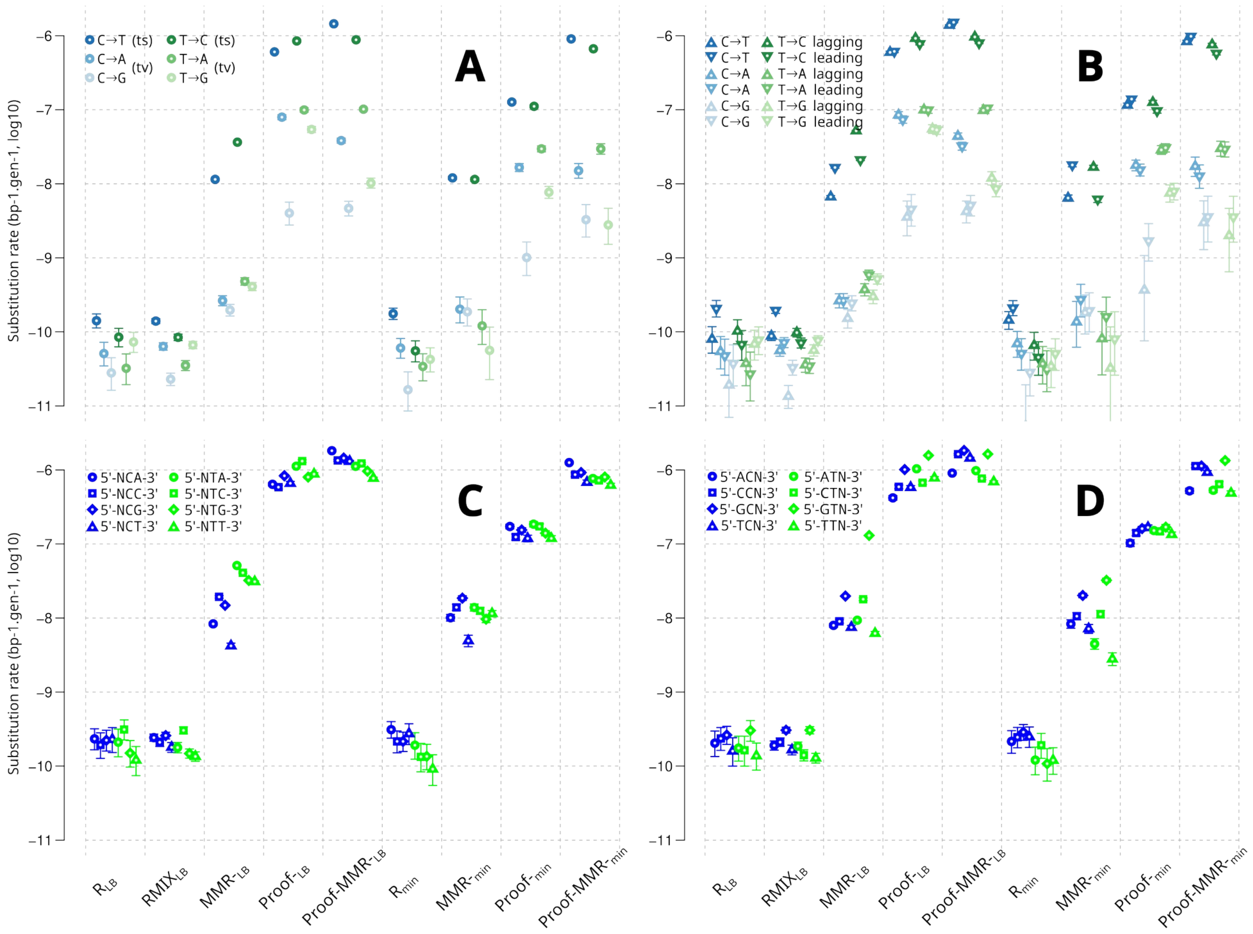

Figure S10. *E. coli* substitution rates in presence or absence of proofreading and MMR and as a function of replication strand and neighboring nucleotides. Rates were recomputed from the results of MA experiments in presence or absence of proofreading and MMR in LB medium and minimal medium (Niccum *et al.*. 1998, Foster *et al.*. 1998). Following Foster et al. 2018, RMIX.LB aggregates data from *E. coli* K12 (wt) and 6 other mutant strains with unaltered substitution rates compared to wt (denoted here R.LB) and MMR-.LB aggregates data from 5 different mutants (for combinations of *mutS*, *mutL* and *mutH* genes); MMR-.min aggregates data from 2 mutants (for *mutS* and *mutL*). Error bars represent the 95% confidence intervals. **A.** Rates of the 6 different types of substitutions. **B.** Effect of orientation with respect to the replication strands (the pyrimidine of the pair determines the strand of a mutation site). **C.** Effect of the 3'-adjacent nucleotide. **D.** Effect of the 5'-adjacent nucleotide.

2.11 Figure S11

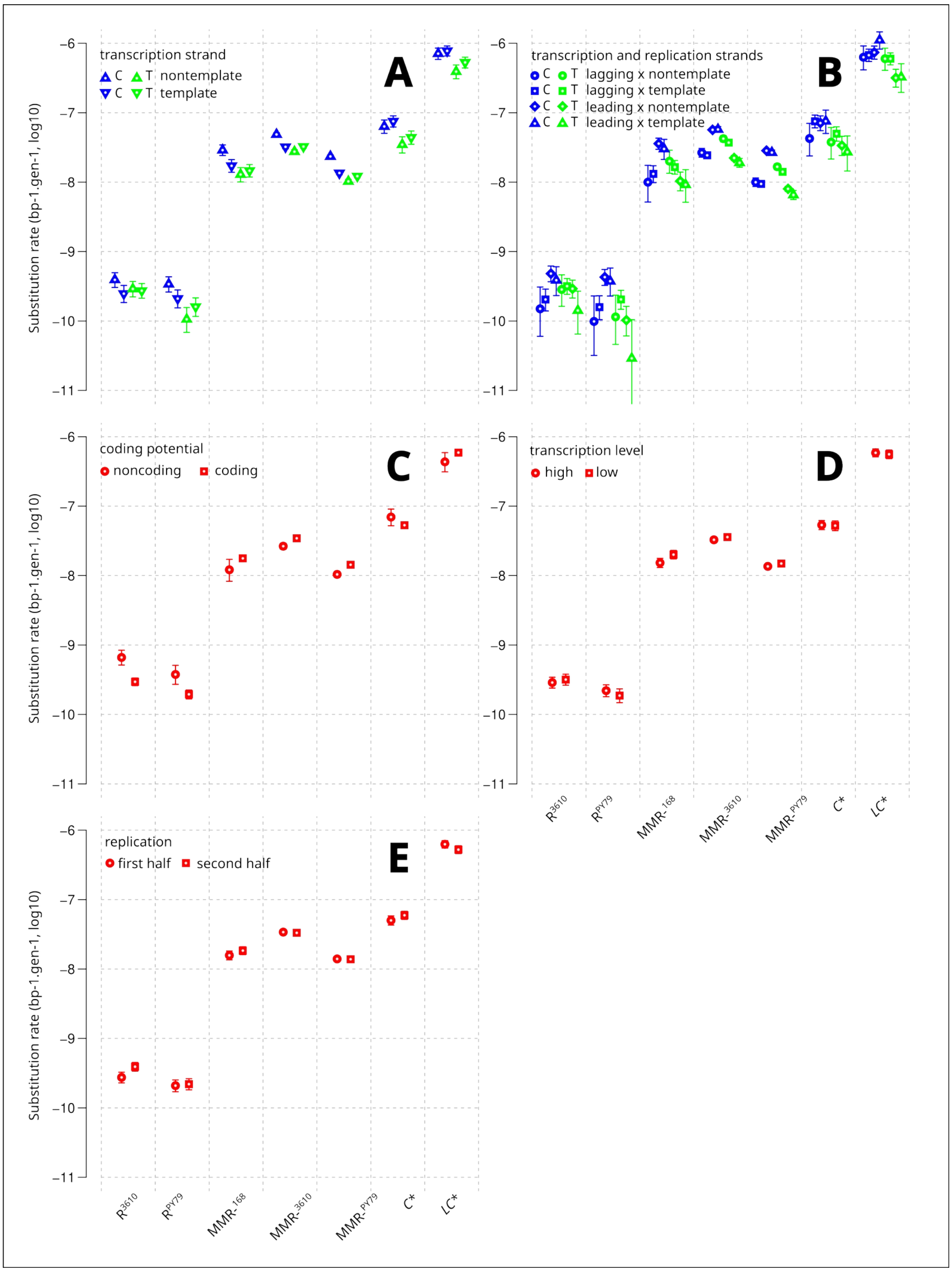

Figure S11. ***B. subtilis*** **substitution rates in different chromosomal contexts with respect to transcription and to replication timing.** Error bars represent the 95% confidence intervals. **A.** Effect orientation with respect to the transcription strand, the pyrimidine of the pair determines the strand of a mutation site. **B.** Combined effects of transcription and replication strands. **C.** Effect of localization in coding or noncoding regions. **D.** Effect of transcription level. **E.** Effect of distance from the origin of replication.

2.12 Figure S12

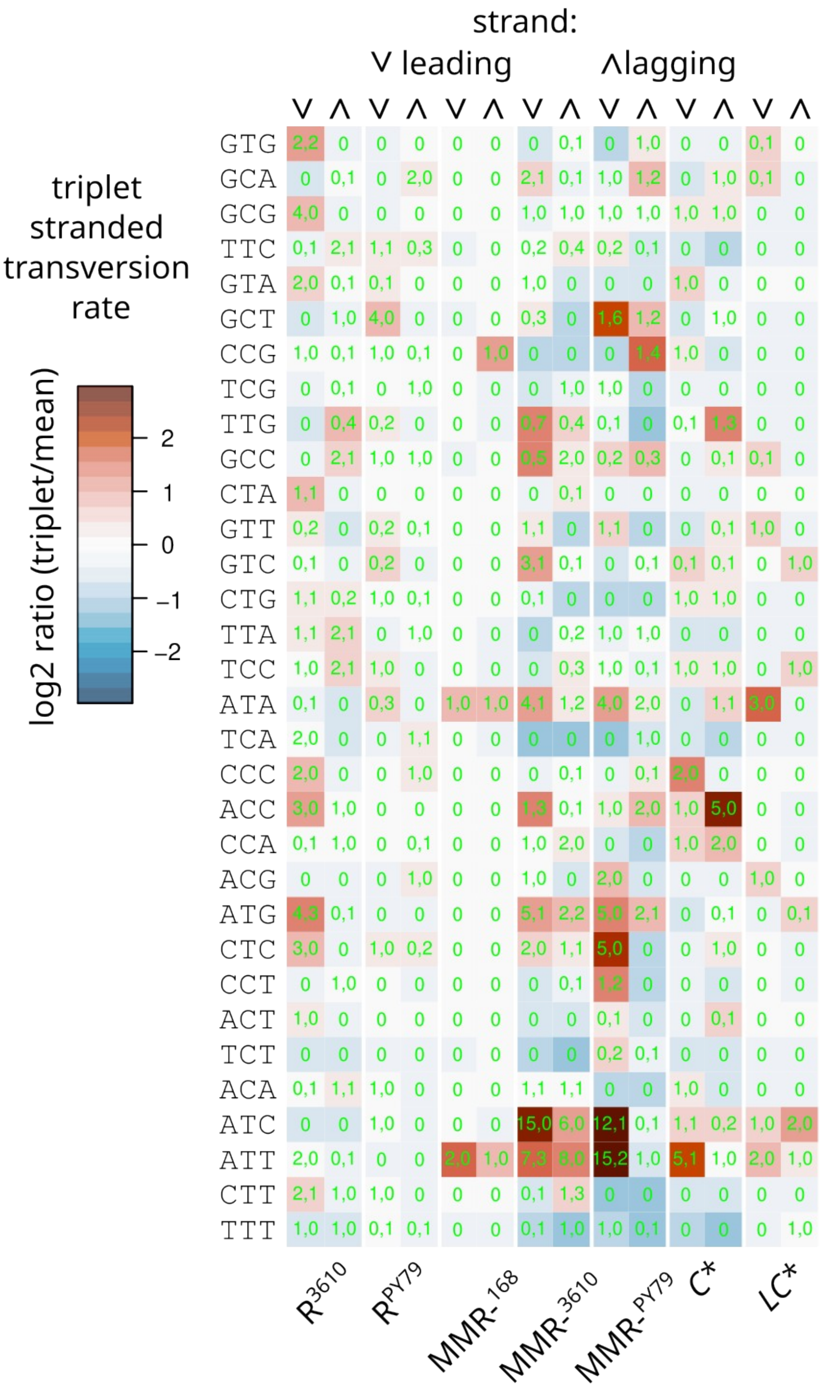

Figure S12. Heatmap representation of the stranded-triplet transversion profiles. Rates were estimated using a Bayesian method involving a log-normal prior and hyperparameters. The numbers of two possible transversions of the focal pyrimidine Y (C or T) are reported in the cells of the heatmap as two numbers separated by a comma (Y→A, Y→ G), written in green. When both numbers are 0, a single number is reported. Triplets are ordered in decreasing order of non-stranded transition rates in R^{3610}.

2.13 Figure S13

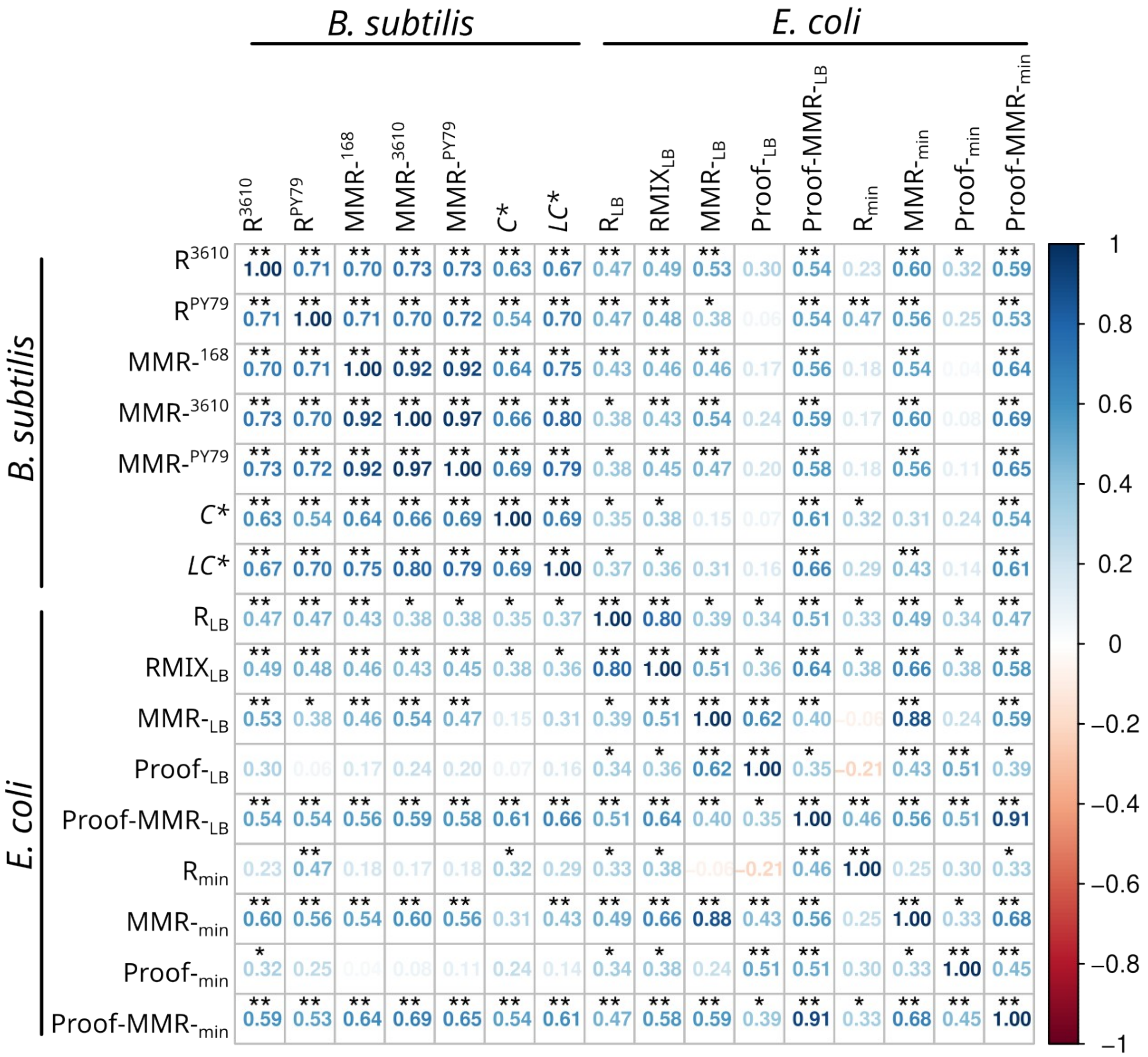

Figure S13. Pairwise correlations between replication-stranded triplet transition rates in different *B. subtilis* and *E. coli* genotypes. The Pearson correlation coefficient between the 64 replication-stranded triplet transition rates (in log-scale) is reported in the matrix and also reflected by the color code. Statistical significance indicated by ** and * for p≤0.001 and p≤0.01.

2.14 Figure S14

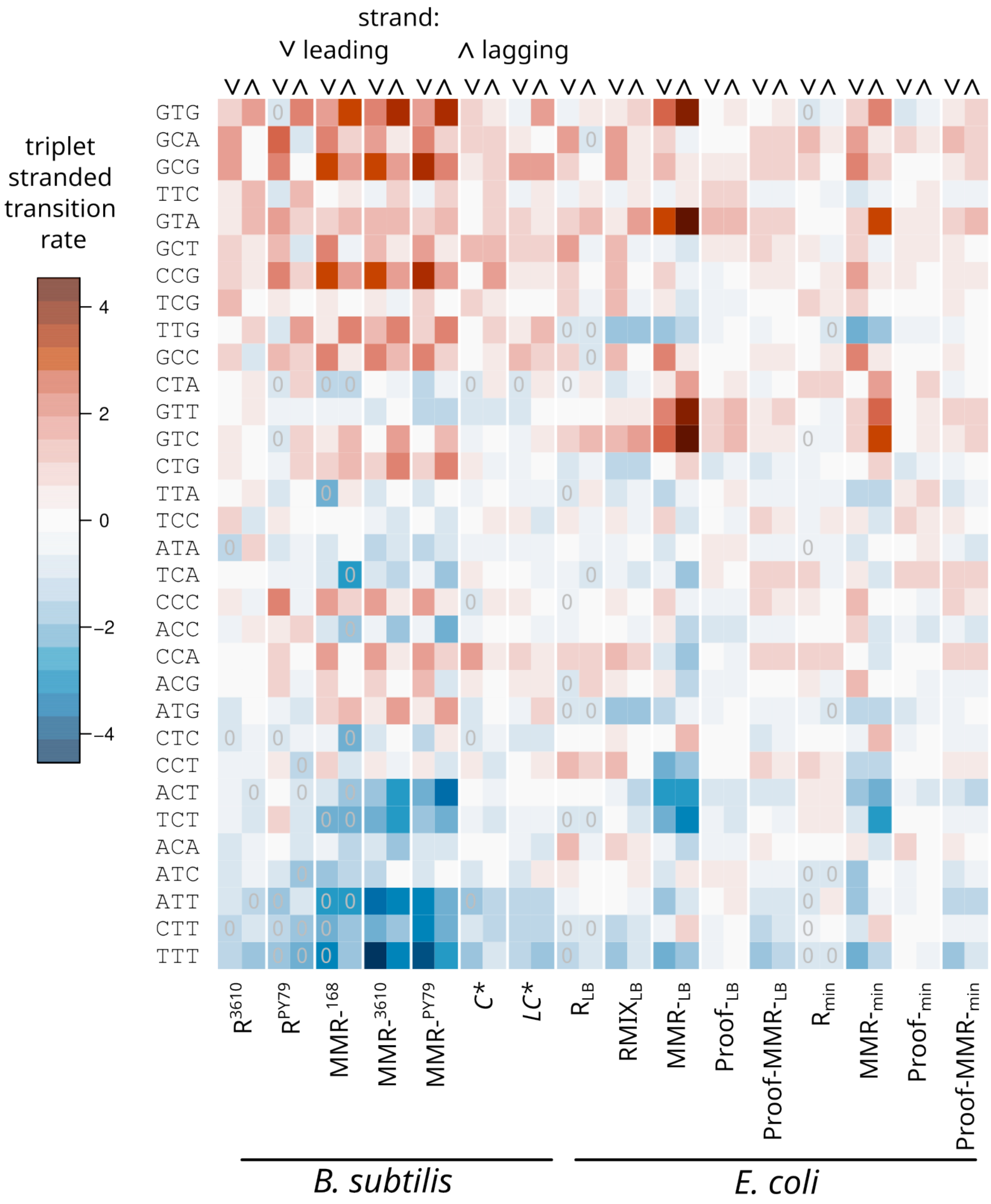

Figure S14. Side-to-side comparison of *B. subtilis* and *E. coli* replication-stranded triplet transition rates. Heatmap representation with triplets are ordered in decreasing order of non-stranded transition rates in *B. subtilis* R^{3610} (two leftmost columns).

2.15 Figure S15

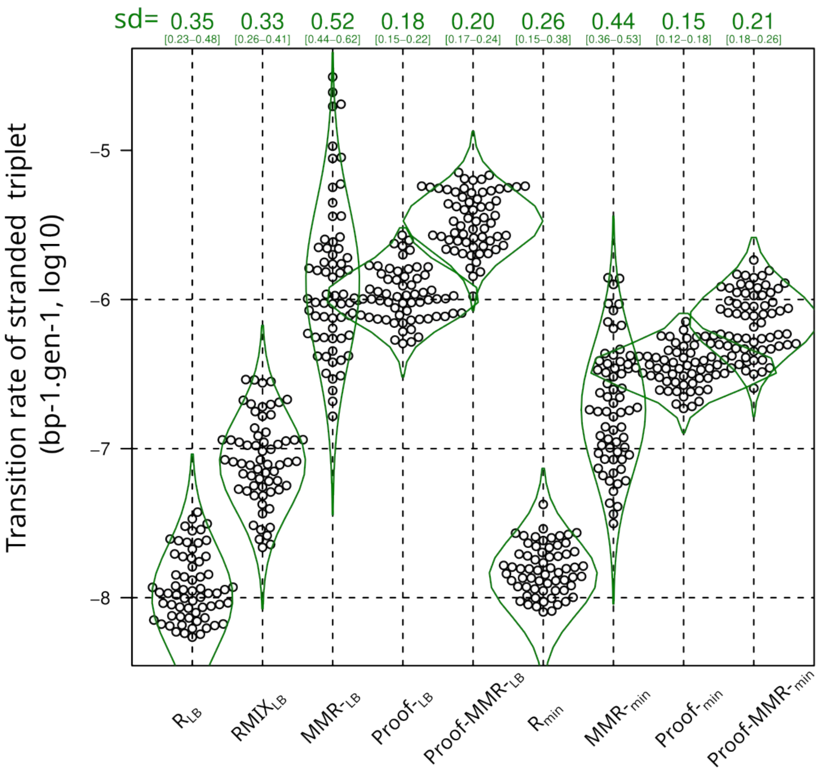

Figure S15. Comparison of the dispersion of stranded-triplet transition rates between *E. coli* genotypes. Beeswarm representation of the distributions of Bayesian estimates of stranded-triplet transition rates, with the Bayesian estimate of their standard deviation in log10-scale reported above along with the corresponding probability density function represented as an envelope (in green).

2.16 Figure S16

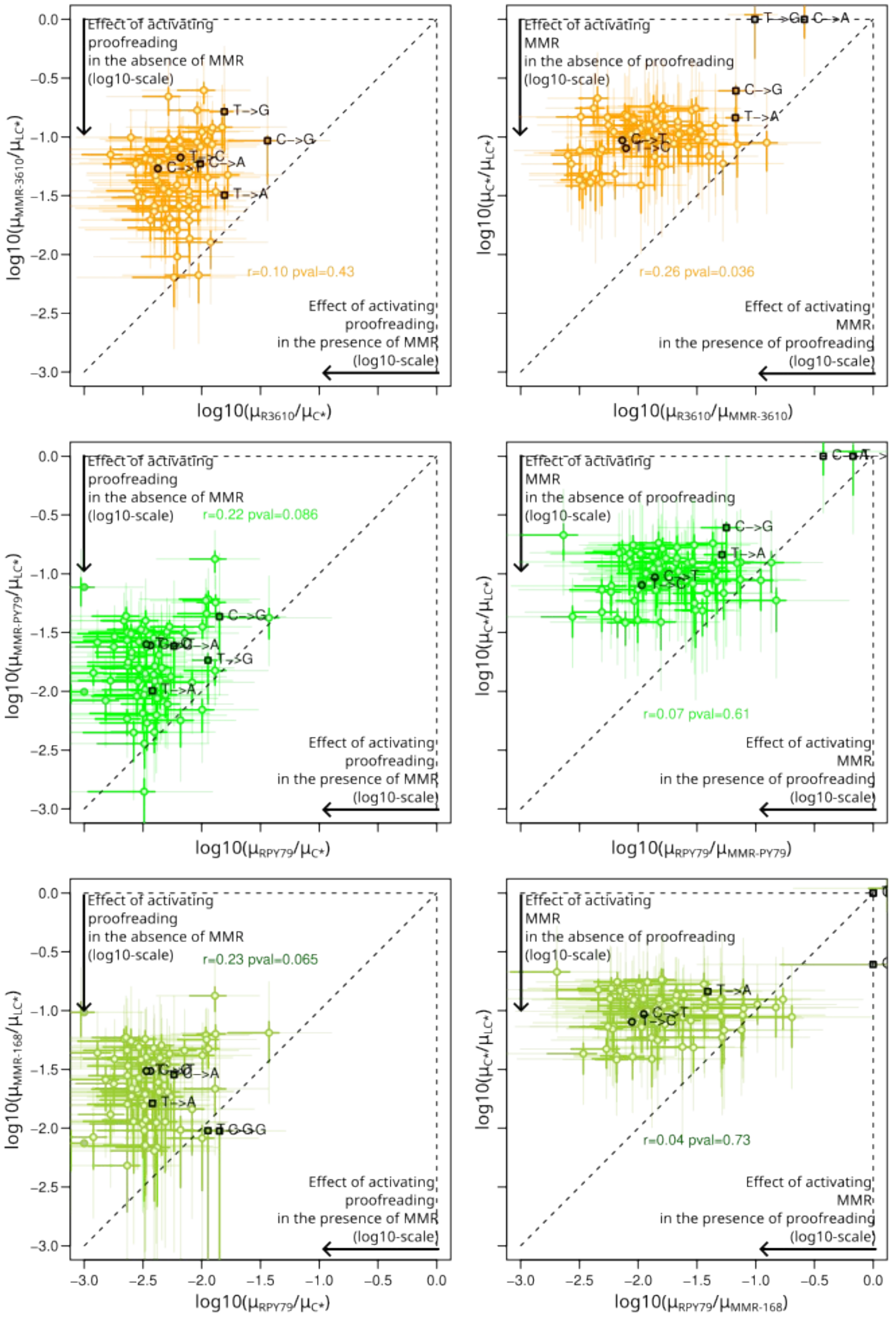

Figure S16. Effects of proofreading and MMR in the presence or absence of the other system, based on different combinations of MMR- and reference genotypes. Left column: effect of proofreading in the presence or absence of MMR. Right column: effect of activating MMR in the presence or absence of proofreading. This figure intends to show the robustness of the pattern seen in main text **Figure 6** (the points tend to be above the first diagonals) with respect to the possible combinations of MMR- and reference genotypes.

2.17 Figure S17

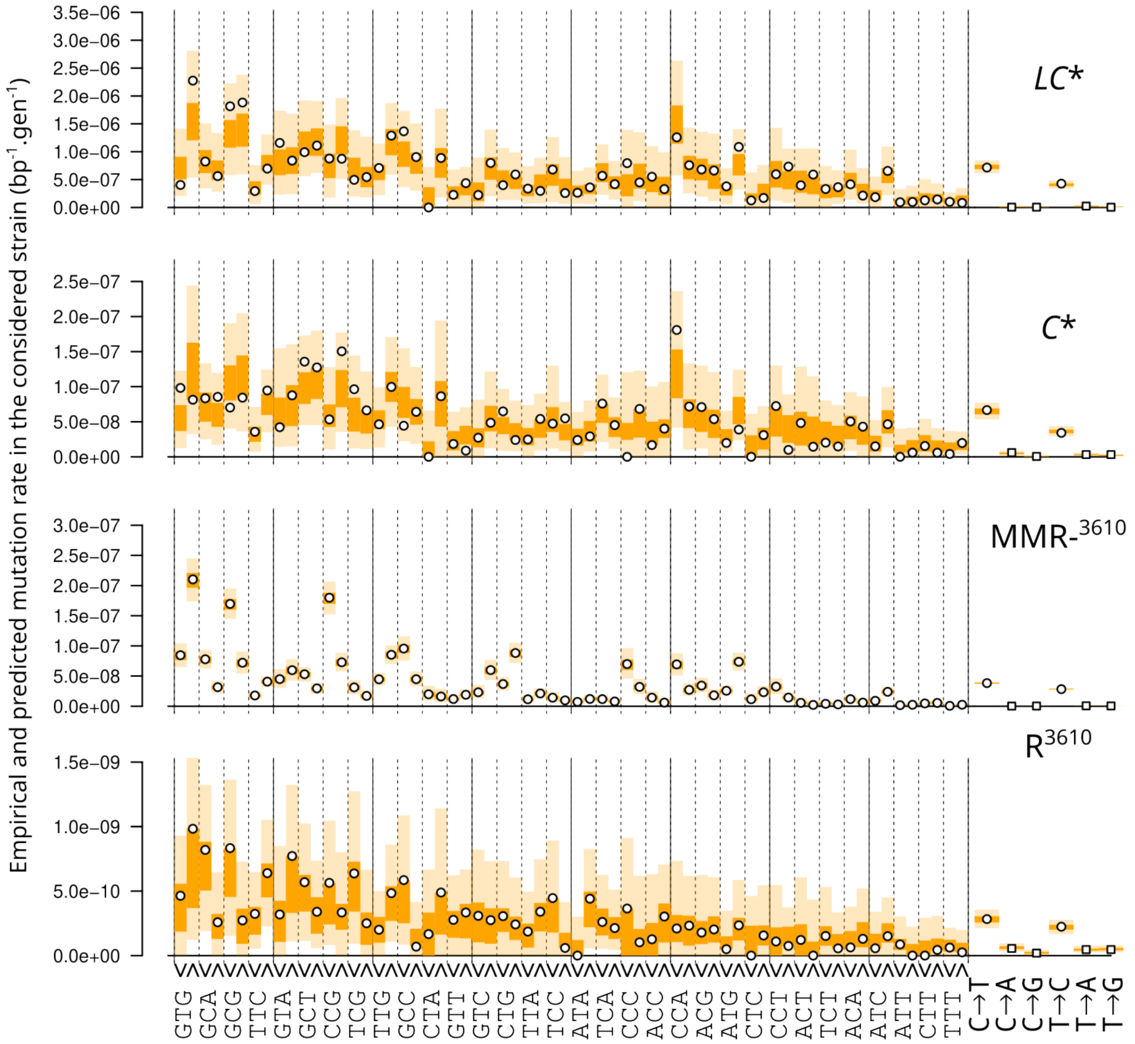

Figure S17. Assessment of the fit of the MMR-saturation model to the experimental data considering the substitution profiles obtained for R^{3610} and MMR^{-3610}. Points represent empirically calculated substitution rates, *i.e.* the number of observed substitutions divided by the number of possible sites in the genome and the number of generations. Colored areas represent the distribution of the empirical rates simulated under the posterior distribution of the model parameters: 50% of the density in the darker areas, 95% if the lighter areas are also considered (all data points are in these intervals). Replication-stranded triplets are ordered in decreasing order of non-stranded empirical substitution rates and then pyrimidine on the leading and lagging strands of replication. The results for the 6 types of substitutions is shown for this combination of reference and MMR- strains in main text **Figure 7C**.

2.18 Figure S18

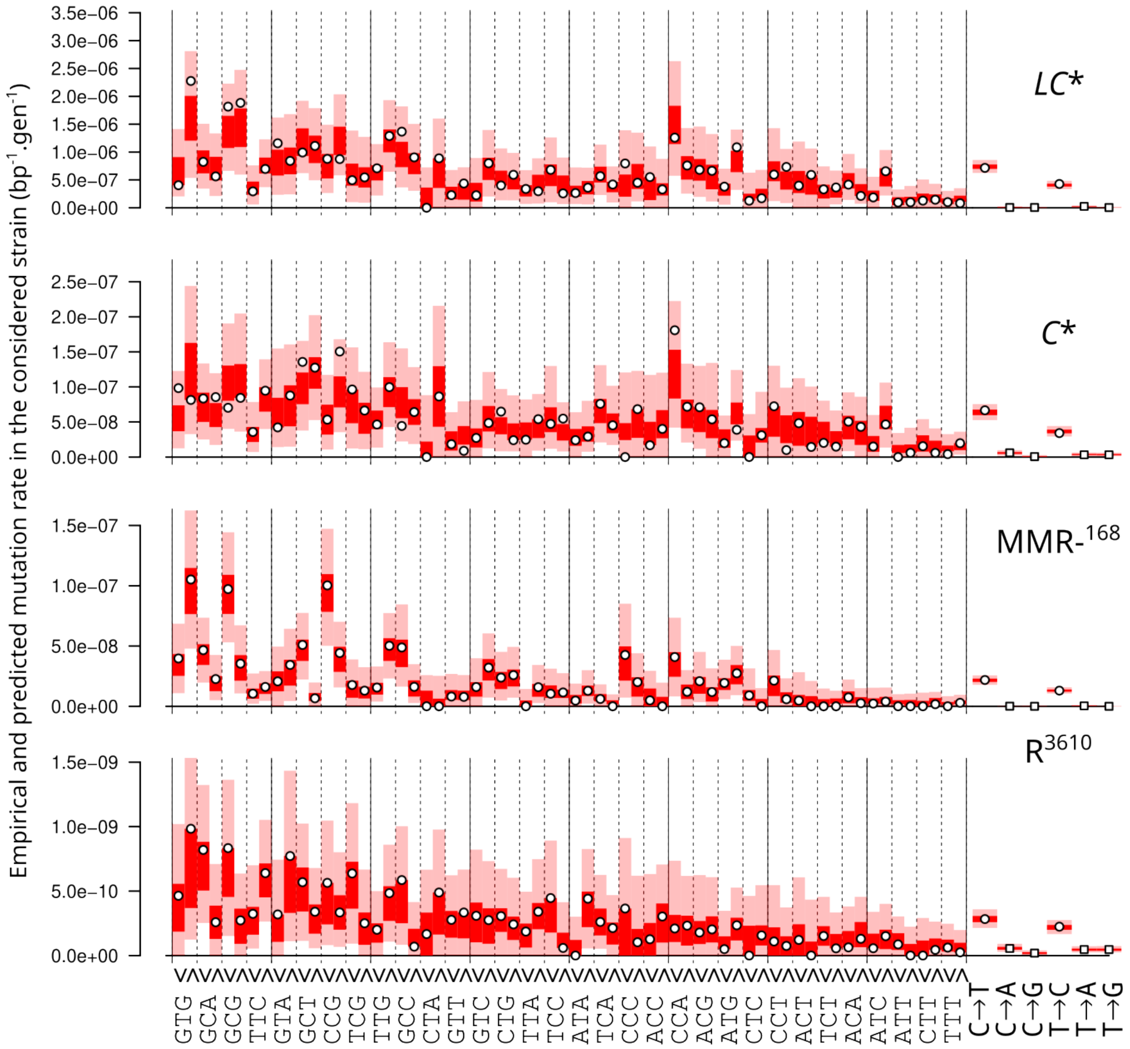

Figure S18. Assessment of the fit of the MMR-saturation model to the experimental data considering the substitution profiles obtained for R^{3610} and MMR-^{168}. Same as **Figure S17** but the data used for MMR-deficient substitution profile is here MMR-^{168} instead of MMR-^{3610}.

2.19 Figure S19

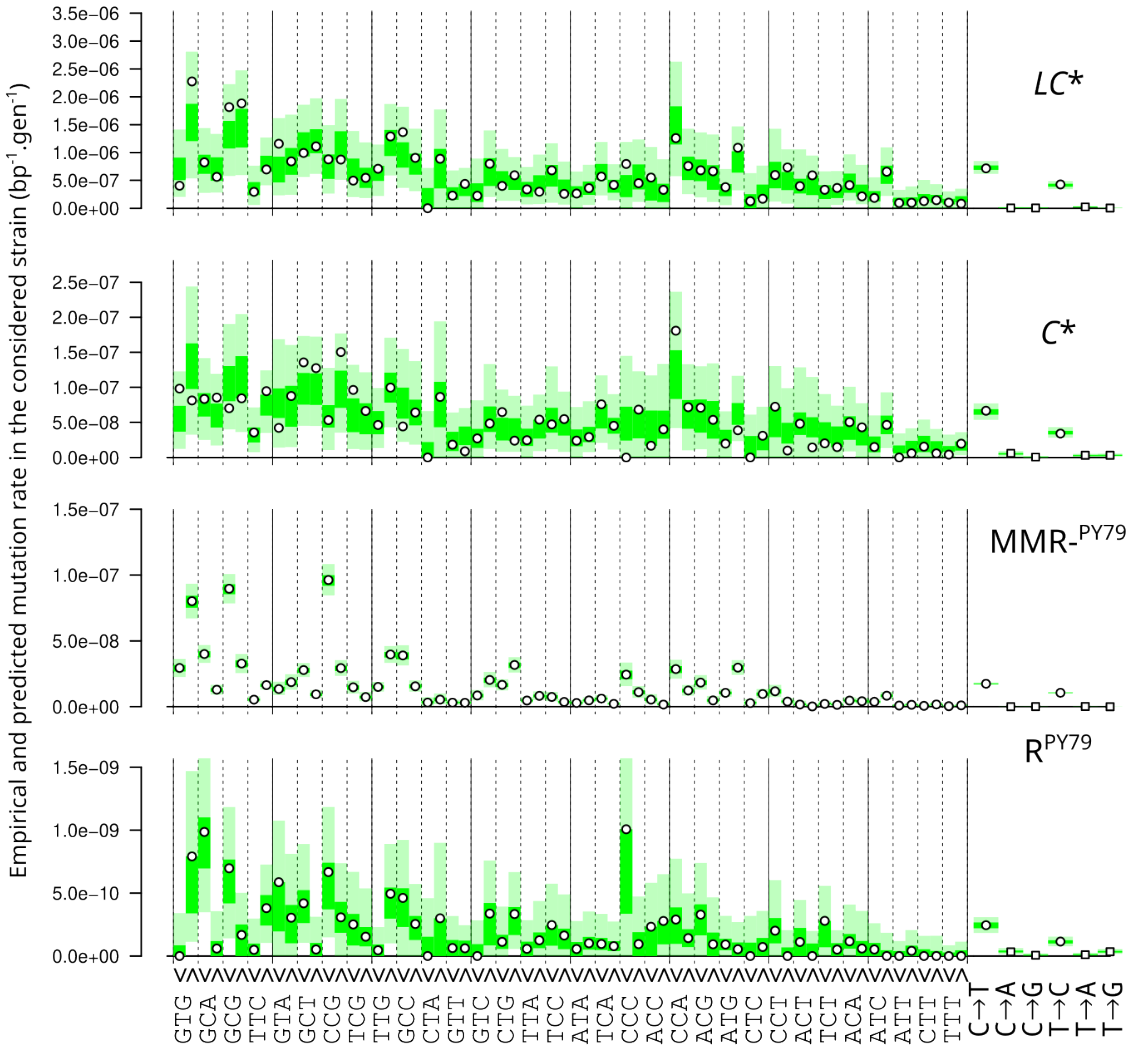

Figure S19. Assessment of the fit of the MMR-saturation model to the experimental data considering the substitution profiles obtained for R^{PY79} and MMR-^{PY79}. Same as **Figure S16 and S17** but the data used for reference and MMR- substitution profiles are here R^{PY79} and MMR-^{PY79} (Schroeder *et al.* 2016).

2.20 Figure S20

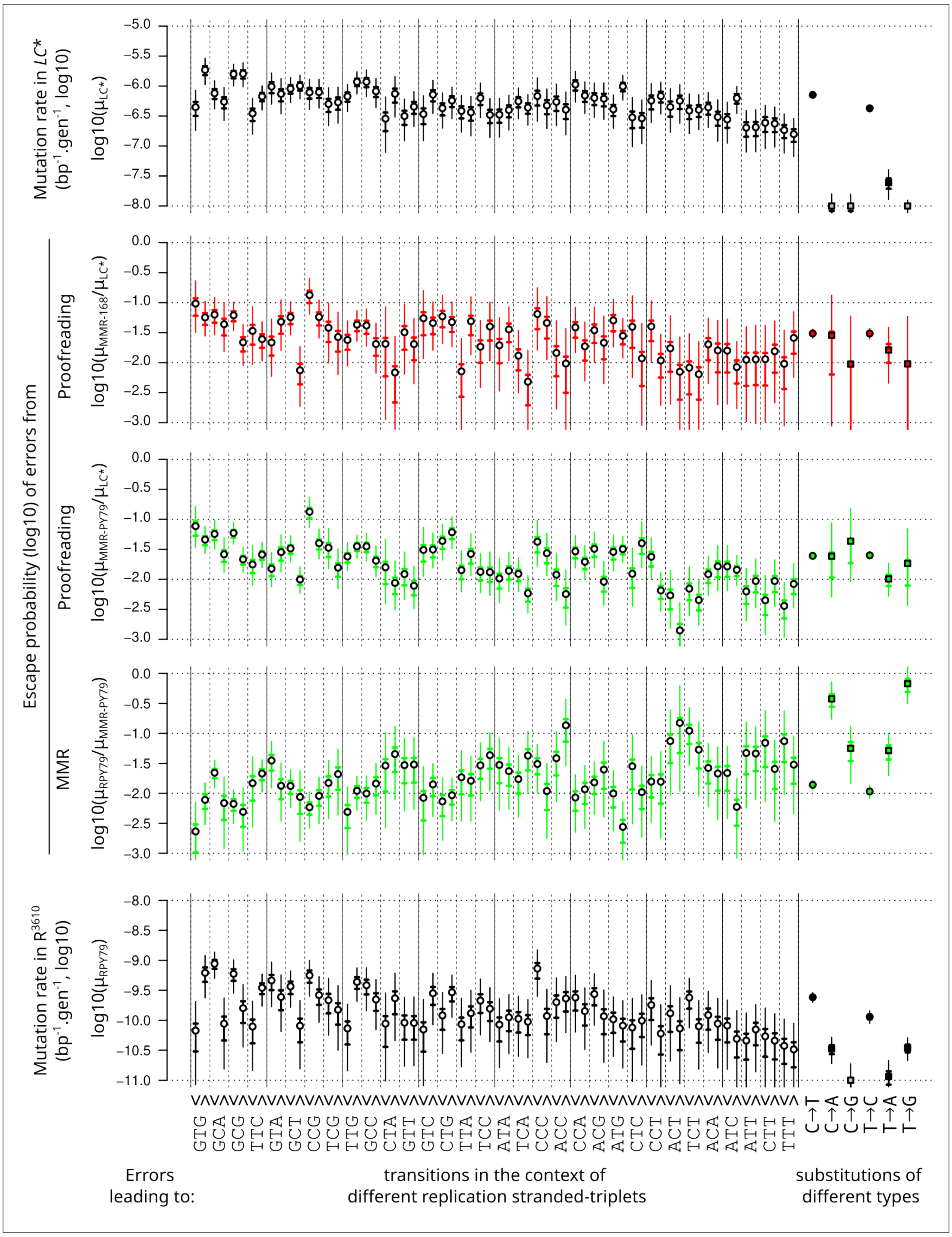

Figure S20. Apparent efficiency of proofreading and MMR across replication-stranded triplets and mutation types, using data from R^{PY79} and MMR-^{PY79}. Same as main text **Figure 8** but using data from R^{PY79} and MMR-^{PY79} in third, fourth and fifth rows (instead of R^{3610} and MMR-^{3610}).

2.21 Figure S21

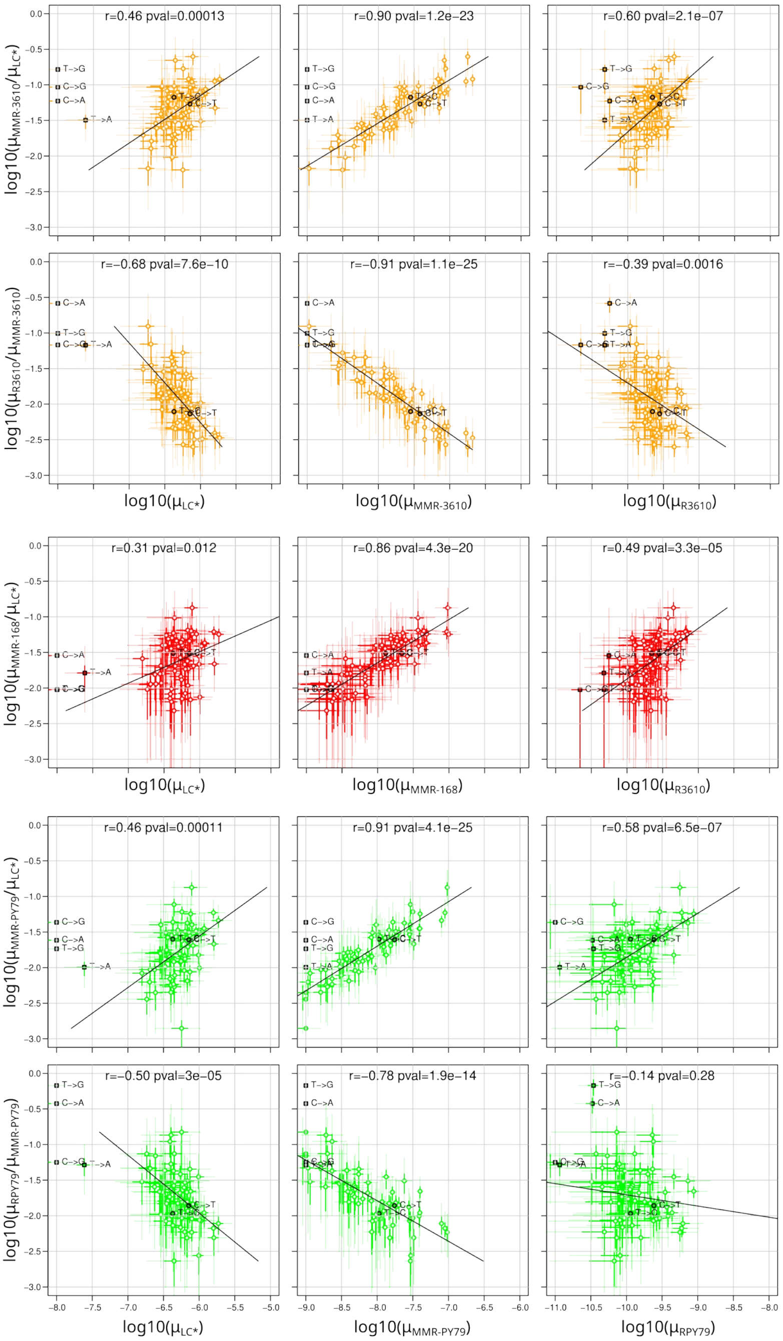

Figure S21. Comparison between proofreading or MMR escape probability and substitution rates in different strains. Each point represents transitions in a replication-oriented triplet or one of the 6 types of substitutions (black circles for transitions, black squares for transversions). Around each point, the 50% and 95% marginal credibility intervals on the horizontal and vertical axes, computed from the quantiles of the posterior distributions, are represented by segments (bold and dark vs. thin and light, respectively). Pearson correlation coefficient computed between escape probability and transition rate (both on logarithmic scale) is reported with the corresponding p-value. The best-fit line obtained by linear regression is shown.

2.22 Figure S22

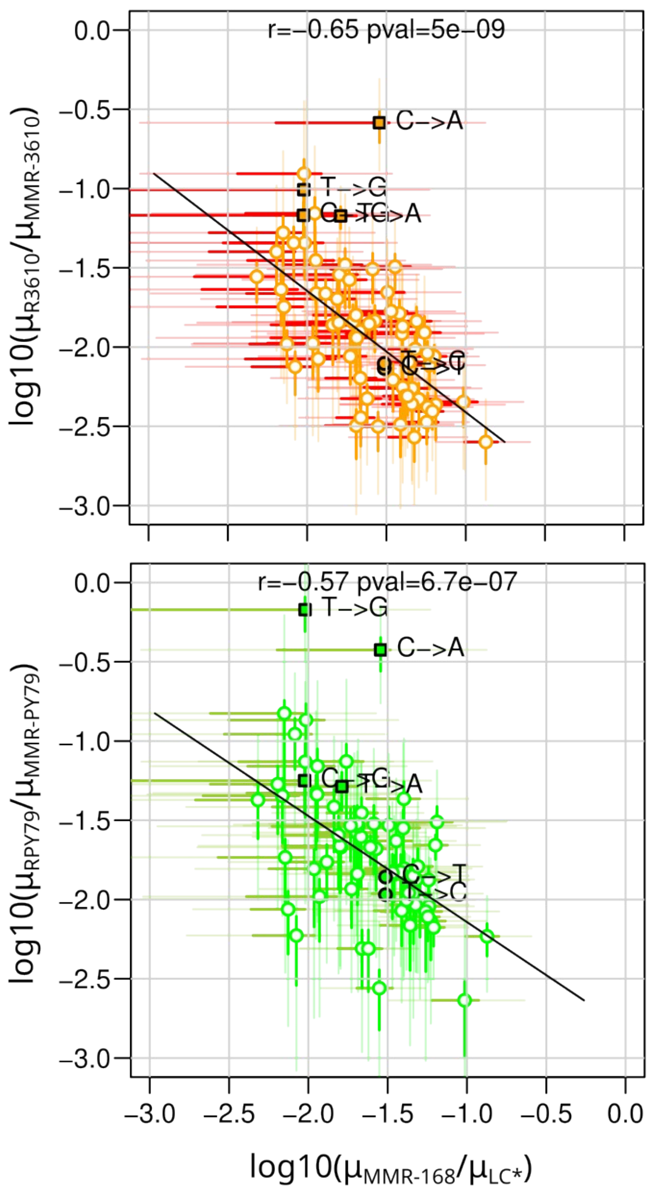

Figure S22. Comparison between apparent escape probability of proofreading and MMR. Each point represents transitions in a replication-oriented triplet or one of the 6 types of substitutions (black circles for transitions, black squares for transversions). Bold and thin bars represent 50% and 95% credibility intervals along each axis). Pearson correlation coefficient computed between apparent MMR (y-axis) and proofreading (x-axis) escape for transitions (both on logarithmic scale) is reported with the corresponding p-value. Apparent MMR escape probability is computed either with R^{3610} and MMR-^{3610} of (Sung *et al.* 2015) of with the R^{PY79} and MMR-^{PY79} of (Schroeder *et al.* 2016). Apparent proofreading escape probability is computed with the data collected in this study (MMR-168 and *LC**).

3 Supplementary Tables

3.1 Table S1

Table S1. Primers used for the construction of strains.

Name	Sequence [a]	Role
P1	5′–GCTCGTTGCACACACCATTT–3′	Amplification of *mutSL* operon – F
P2	5′–TGAAAAAGGCCCTTCCCCAG–3′	Amplification of *mutSL* operon – R
P3	5′–ACTAAGCTTAATTGTTATCCGCTCA–3′	Linearization pDR111 – F
P4	5′–GCATGCAAGCTAATTCGGTGG–3′	Linearization pDR111 - R
P5	5′–GGATAACAATTAAGCTTAGTCGGGAAGGAGGAACTACTGTGGCAAAAGTCATCCAACTGT–3′	Amplification of *mutL* for Gibson assembly in pDR111 – F
P6	5′–CCGAATTAGCTTGCATGCTCACACGAACAGGGAGCAAA–3′	Amplification of *mutL* for Gibson assembly in pDR111 -R
P7	5′–*CGTCAAAGAATTGGTGGAA**C**ATGCGATCGACGCTGACAGCA*CAGTCATTG–3′	Introduction of N34H mutation on *mutL* – F
P8	5′–*TGCTGTCAGCGTCGATCGCAT**G**TTCCACCAATTCTTTGACG*ACTGAGGCGG –3′	Introduction of N34H mutation on *mutL* – R
P9	5′–GGATAACAATTAAGCTTAGTTTTAGGGAGGGATACTGTCT–3′	Amplification of *polC** for Gibson assembly in pDR111 – F
P10	5′–CCGAATTAGCTTGCATGCCCTAAGCAAGTGACAGAAACT–3′	Amplification of *polC** for Gibson assembly in pDR111 – R
P11	5′–CTACATCACGCGTTTGAAC–3′	Amplification of *mutL** for Gibson assembly with *polC** - R
P12	5′–TGTTCAAACGCGTGATGTAGTTTTAGGGAGGGATACTGTCT–3′	Amplification of *polC** for Gibson assembly with mutL* - F

[a] Regions used for the HiFi DNA assembly protocol are underlined. The single nucleotide mutations used to build the *mutL*(N34H) variant is indicated in bold. The sequences in primers P7 and P8 that allow for the assembly by PCR are indicated in italics.

3.2 Table S2

Table S2. Detailed results of fluctuation assays.

Strain	[IPTG] (µM)	#cultures[a]	#zeros[b]	mean M[c]	V[d] (mL)	mean C[e] ($\times10^7$)	sd C[f] ($\times10^7$)	MLE m[g]	RifR mutation rate[h]
R^{168}	0	96	84	0.14	1.0	12.95	6.66	0.13	9.74×10^{-10}
R^{168}	100	96	76	0.25	0.2	7.99	7.60	0.21	2.68×10^{-9}
ΔS	0	16	0	37.75	1.0	12.95	6.66	9.64	7.54×10^{-8}
ΔL	0	16	0	50.69	1.0			11.79	9.10×10^{-8}
*L**	0	8	6	0.25	1.0	11.82	5.25	0.25	2.12×10^{-9}
	5	9	7	0.22	0.2	4.48	2.25	0.22	4.85×10^{-9}
	20	8	0	36.63	1.0	11.82	5.25	11.21	9.48×10^{-8}
	50	9	0	27.00	0.2	3.60	2.50	8.14	2.26×10^{-7}
	100	8	0	506.38	1.0	11.82	5.25	43.19	3.66×10^{-7}
	500	8	0	191.38	1.0			40.88	3.46×10^{-7}
*C**	0	9	6	0.33	0.2	4.58	2.25	0.33	7.27×10^{-9}
	5	9	7	1.67	0.2			0.27	5.81×10^{-9}
	20	9	0	8.67	0.2			1.76	3.83×10^{-8}
	50	9	0	60.56	0.2			16.41	3.58×10^{-7}
	100	9	0	228.22	0.2			45.73	9.98×10^{-7}
	500	9	0	459.67	0.2			82.65	1.80×10^{-6}
*LC**	0	9	6	0.56	0.2	3.60	2.50	0.39	1.09×10^{-8}
	5	9	5	4.56	0.2			0.55	1.52×10^{-8}
	20	9	0	32.44	0.2			9.99	2.77×10^{-8}
	50	9	0	1166.00	0.2			178.09	4.94×10^{-6}
	100	9	0	1404.89	0.2			208.13	5.78×10^{-6}
	500	9	0	1351.56	0.2			202.25	5.61×10^{-6}

[a] Number of cultures for RifR determination. [b] Number of samples without any RifR mutants. [c] Mean number of mutants per culture. [d] Culture volume (mL), the dilution factor used to estimate the total number of cells was 20×10^5 for 1 mL cultures 2×10^5 for 0.2 mL cultures. [e-f] Mean and standard deviation of the final number of cells (calculated by day) ($\times10^7$). [g] MLE of the number of mutations per culture. [h] Rate of emergence of the RifR phenotype estimated from the number of mutations and the number of cells per culture.

3.3 Table S3

Table S3. Induction of *mutL and *polC** by IPTG as measured by RNA-Seq.**

Str[a]	[IPTG] (μM)	R[b]	*mutL+L** (fpkm)[c]	mutL* N34H[d]	*polC+C** (fpkm)[c]	*polC** G430E[d]	*polC** S621N[d]
R[168]	0	1	104.3	0.00 (0.00-0.02)	159.5	0.00 (0.00-0.02)	0.00 (0.00-0.03)
R[168]	0	2	106.1	0.00 (0.00-0.03)	159.5	0.00 (0.00-0.01)	0.00 (0.00-0.02)
R[168]	100	1	96.5	0.00 (0.00-0.02)	144.5	0.00 (0.00-0.02)	0.00 (0.00-0.03)
R[168]	100	2	116.7	0.00 (0.00-0.02)	171.5	0.00 (0.00-0.01)	0.00 (0.00-0.02)
*L**	100	1	7,020.1	0.99 (0.99-1.00)	134.4	0.00 (0.00-0.05)	0.00 (0.00-0.07)
*L**	100	2	7,234.1	0.99 (0.98-0.99)	108.2	0.00 (0.00-0.03)	0.00 (0.00-0.04)
*C**	100	1	72.3	0.00 (0.00-0.04)	4,471.8	0.98 (0.97-0.98)	0.97 (0.97-0.98)
*C**	100	2	74.5	0.00 (0.00-0.05)	3,905.8	0.97 (0.97-0.98)	0.98 (0.98-0.99)
*LC**	0	1	148.8	0.35 (0.28-0.42)	197.0	0.18 (0.13-0.23)	0.25 (0.18-0.32)
*LC**	0	2	98.6	0.29 (0.22-0.38)	123.7	0.19 (0.13-0.27)	0.28 (0.19-0.40)
*LC**	100	1	3,601.8	0.98 (0.98-0.99)	3,084.8	0.97 (0.97-0.98)	0.96 (0.95-0.97)
*LC**	100	2	5,905.3	0.98 (0.98-0.98)	5,356.4	0.96 (0.96-0.97)	0.97 (0.96-0.97)

[a] Tested strain. [b] RNA-Seq biological replicate. [c] Quantification of the amount of transcript for this gene (wild-type and mutant allele) in fpkm (fragments per kilobase of transcript per million mapped reads). [d] Proportion of RNA-Seq reads carrying the mutation out of the reads overlapping this position.

3.4 Table S5

Table S5. Aggregated numbers of indels, indel rate and proportion of insertions for each investigated strain.

Strain	Indels[a]		Indel rate [95% CI]	Proportion of insertions [95% CI]
	insertions	deletions		
R^{168}	0	3	2.1e-10 [0.4-6.1e-10]	0.00 [0.00-0.71]
R^{3610}	20	53	7.7e-11 [6.0-9.6e-11]	0.27 [0.18-0.39]
R^{PY79}	17	18	3.4e-11 [2.4-4.7e-11]	0.49 [0.31-0.66]
ΔS	31	33	7.8e-09 [6.0-10e-09]	0.48 [0.36-0.61]
ΔL	16	58	9.1e-09 [7.1-11e-09]	0.22 [0.13-0.33]
*L**	24	53	9.4e-09 [7.4-12e-09]	0.31 [0.21-0.43]
$MMR\text{-}^{168}$	71	144	8.8e-09 [7.6-10e-09]	0.33 [0.27-0.40]
$MMR\text{-}^{3610}$	452	267	5.0e-09 [4.6-5.4e-09]	0.63 [0.59-0.66]
$MMR\text{-}^{PY79}$	543	415	2.4e-09 [2.3-2.6e-09]	0.57 [0.53-0.60]
*C**	52(2)	35(0)	1.2e-08 [1.0-1.5e-08]	0.60 [0.49-0.70]
*LC**	99(125)	50(90)	1.7e-07 [1.4-2.0e-07]	0.66 [0.58-0.74]

[a] Only short indels (length ≤ 2 bp) are counted. Between parentheses: number of indels in time intervals with decreased mutation rates (discarded from the analysis).

3.5 Table S6

Table S6. Mutations in MA-lines on the inserted regions for *L**, *C** and *LC** strains.

MA line	MA-step[a]	Position[b]	Region[c]	Ref.	Alt[d]	Gene[e]	AA	PolC domain or position in insert[f]	Consequence on coding regions[g]
*L** 1	11	3244	native	C	-1T	*mutL*	361+	-	Frameshift
*C** 3	11	4683	insert	C	T	*polC*	618	Exo - PHP domains	Nonsyn. (GCC→ACC)
*C** 3	11	4477	insert	G	A	*polC*	686	-	Syn. (TTC→TTT)
*C** 4	11	6296	insert	G	A	*polC*	80	N-ter domain	Nonsyn. (TCT→TTT)
*C** 4	11	8014	insert	T	-1A	*spec*^{R}	255+	-	Frameshift
*LC** 1	11	404	insert	C	T	Inter.	-	amyE gene	-
*LC** 1	11	696	insert	G	A	Inter.	-	amyE gene	-
*LC** 1	1	8592	insert	T	+1A	Inter.	-	50 nt upstream mutL* Phs	-
*LC** 2	3	5325	insert$	C	T	*polC*	404	PHP - Exo domain	Nonsyn. (GGC→AGC)
*LC** 2	6	6217	insert$	C	T	*polC*	106	-	Syn. (CAG→CAA)
*LC** 2	6	2455	native$	G	A	*polC*	1360	-	Syn. (TCC→TCT)
*LC** 2	6	1932	insert	T	G	Inter.	-	lacI promoter	-
*LC** 3	6	4251	insert$	T	C	*polC*	762	PHP domain	Nonsyn. (AAT→GAT)
*LC** 3	11	5345	native$	T	C	*polC*	397	PHP - Exo domain	Nonsyn. (GAA→GGA)
*LC** 4	6	6399	insert	A	G	*polC*	46	N-ter domain	Nonsyn. (TGG→CGG)
*LC** 4	3	4245	native	G	A	*polC*	764	PHP domain	Nonsyn. (CAT→TAT)

[a] MA-step of the first detection. [b] Position of the mutation as seen when the sequencing read is mapped to the insert (see **Figure S2**) [c] Mutations detected in *mutL* and *polC* could have occurred either in the native allele or in the mutant allele located in the insert. For these two genes, the information in this column is derived from the frequency of the mutation in the reads (see **Figure S9,** experimentally verified by PCR amplification and sequencing when indicated by the symbol $). [d] Observed substitution, deletion (-) or insertion (+). [e] Inter. for intergenic. [f] As defined in Evans *et al.* (2008), the 3'-5' exonuclease domain of PolC spans amino acids 412 to 617. It is flanked by the polymerase and histidinol phosphatase (PHP) domains, which extend from amino acids 342 to 412 and 617 to 829, respectively. Regions downstream of amino acid 829 are associated with the core polymerase function. [g] Syn. for synonymous Nonsyn. for nonsynonymous.

Supplementary References

References not cited in the main text.

Gelman, A. G. (2006). Prior distributions for variance parameters in hierarchical models (Comment on Article by Browne and Draper). Bayesian Analysis, 1(3):515–534.

Gelman, A. G. and Rubin, D. B. (1992). Inference from iterative simulation using multiple sequences. Statistical Science, 7(4):457–511.

Gelman AG, Carlin JB, Stern HS, Dunson DB, Vehtarti A and Rubin DB, Bayesian data analysis, Third. Boca Raton, FL: CRC press, 2014.

Lujan SA, Clark AB, Kunkel TA. 2015. Differences in genome-wide repeat sequence instability conferred by proofreading and mismatch repair defects. *Nucleic Acids Res* **43**: 4067-4074.

Streisinger G, Okada Y, Emrich J, Newton J, Tsugita A, Terzaghi E, Inouye M. 1966. Frameshift mutations and the genetic code. This paper is dedicated to Professor Theodosius Dobzhansky on the occasion of his 66th birthday. *Cold Spring Harb Symp Quant Biol* **31**: 77-84.
